# HGF/c-Met pathway facilitates the perineural invasion of pancreatic cancer by activating the mTOR/NGF axis

**DOI:** 10.1038/s41419-022-04799-5

**Published:** 2022-04-21

**Authors:** Tao Qin, Ying Xiao, Weikun Qian, Xueni Wang, Mengyuan Gong, Qiqi Wang, Rui An, Liang Han, Wanxing Duan, Qingyong Ma, Zheng Wang

**Affiliations:** 1 Department of Hepatobiliary Surgery, The First Affiliated Hospital of Xi’an Jiaotong University, Xi’an, China; 2 Centre for Pancreatic Diseases of Xi’an Jiaotong University, Xi’an, China; 3 Key Laboratory of Environment and Genes Related to Diseases, Xi’an Jiaotong University, Xi’an, China

**Keywords:** Pancreatic cancer, Cancer microenvironment

## Abstract

Perineural invasion (PNI) is a pathologic feature of pancreatic cancer and is associated with poor outcomes, metastasis, and recurrence in pancreatic cancer patients. However, the molecular mechanism of PNI remains unclear. The present study aimed to investigate the mechanism that HGF/c-Met pathway facilitates the PNI of pancreatic cancer. In this study, we confirmed that c-Met expression was correlated with PNI in pancreatic cancer tissues. Activating the HGF/c-Met signaling pathway potentiated the expression of nerve growth factor (NGF) to recruit nerves and promote the PNI. Activating the HGF/c-Met signaling pathway also enhanced the migration and invasion ability of cancer cells to facilitate cancer cells invading nerves. Mechanistically, HGF/c-Met signaling pathway can active the mTOR/NGF axis to promote the PNI of pancreatic cancer. Additionally, we found that knocking down c-Met expression inhibited cancer cell migration along the nerve, reduced the damage of the sciatic nerve caused by cancer cells and protected the function of the sciatic nerve in vivo. Taken together, our findings suggest a supportive mechanism of the HGF/c-Met signaling pathway in promoting PNI by activating the mTOR/NGF axis in pancreatic cancer. Blocking the HGF/c-Met signaling pathway may be an effective target for the treatment of PNI.

## Introduction

Pancreatic cancer is one of the most fatal malignant tumors, with a mortality rate close to the incidence rate. No specific symptoms are observed in the early stage of pancreatic cancer, and diagnosing at an early stage is difficult. Most pancreatic cancer patients are in the advanced stage at diagnosis because of invasion and metastasis [1]. The 5-year survival rate for pancreatic cancer patients is approximately 10% [2]. Surgical resection is the only cure for pancreatic cancer, but the recurrence rate is extremely high. The 5-year survival rate of patients undergoing surgical resection is only 25% [3]. Exploring the mechanism of occurrence and development of pancreatic cancer is of great significance for the prevention and treatment of pancreatic cancer. Evidence indicates that perineural invasion (PNI) is associated with poor outcomes and is an important predictor of recurrence in pancreatic cancer [4].

PNI is a pathological process that eventually leads to cancer cells invading peripheral nerves. PNI was first reported in head and neck tumors and was subsequently researched in various tumors, including gastric cancer [5], breast cancer [6], prostate cancer [7]. Liebig presented a definition of PNI as cancer cells closely contacting the nerve and surrounding at least 33% of the nerve periphery or invading any of the three layers of the neurolemma structure [8]. PNI is a crucial pathological hallmark of pancreatic cancer and pancreatic cancer has the highest incidence of PNI, approximately 70–100%, among all solid tumors [9]. PNI has an unfavorable effect on overall survival and is a dismal prognostic factor that doubles the risk of local recurrence in pancreatic cancer when regarding disease-free survival and progression-free survival [10].

Cellular and molecular mechanisms have been proposed to be involved in PNI. Schwann cells can migrate inwards to the tumor before PNI and may contribute to the PNI initiation process [11]. In our previous study, activating pancreatic stellate cells can enhance the interaction between cancer cells and the dorsal root ganglia (DRG) [12, 13]. Additionally, neurotrophins, including nerve growth factor (NGF), brain-derived neurotrophic factor (BDNF), glial cell line-derived neurotrophic factor (GDNF), and chemokines and neurotransmitters exert various effects in regulating PNI in pancreatic cancer [14]. However, the mechanism of PNI remains poorly understood. A more comprehensive understanding of the crosstalk between cancer cells and nerves is a potential strategy for cancer therapy.

Hepatocyte growth factor (HGF) is mainly produced by stromal cells of mesenchymal origin. c-Met (hepatocyte growth factor receptor, HGFR) is a member of the receptor tyrosine kinase family and is mainly expressed in epithelial cells [15]. HGF is the sole ligand for c-Met, and HGF binding with c-Met can lead to c-Met phosphorylation, which activates several intracellular signaling pathways, including MAPK, STAT3, and PI3K/AKT. These pathways regulate the proliferation, invasion, and migration of cancer cells [15]. In pancreatic cancer, the c-Met receptor is expressed by cancer cells and endothelial cells [15]. A recent study found that c-Met is an important hub gene of pancreatic cancer by RNA-seq and microarray dataset analyses [16]. The HGF levels in serum have been reported to correlate with pancreatic cancer progression in patients [17]. The HGF/c-Met pathway plays a key role in the progression of metastasis by influencing cell-cell junctions, supporting cytoskeletal remodeling, and controlling integrin function, among other functions [18]. Our previous study showed that PSCs facilitate perineural invasion of pancreatic cancer via the HGF/c-Met pathway [13].

However, the underlying mechanisms by which the HGF/c-Met pathway promotes PNI in pancreatic cancer remain unknown. In this study, we further explored the possible mechanism by which the HGF/c-Met pathway promotes PNI in pancreatic cancer.

## Results

### c-Met is overexpressed and associated with PNI in pancreatic cancer

**Fig. 1:**
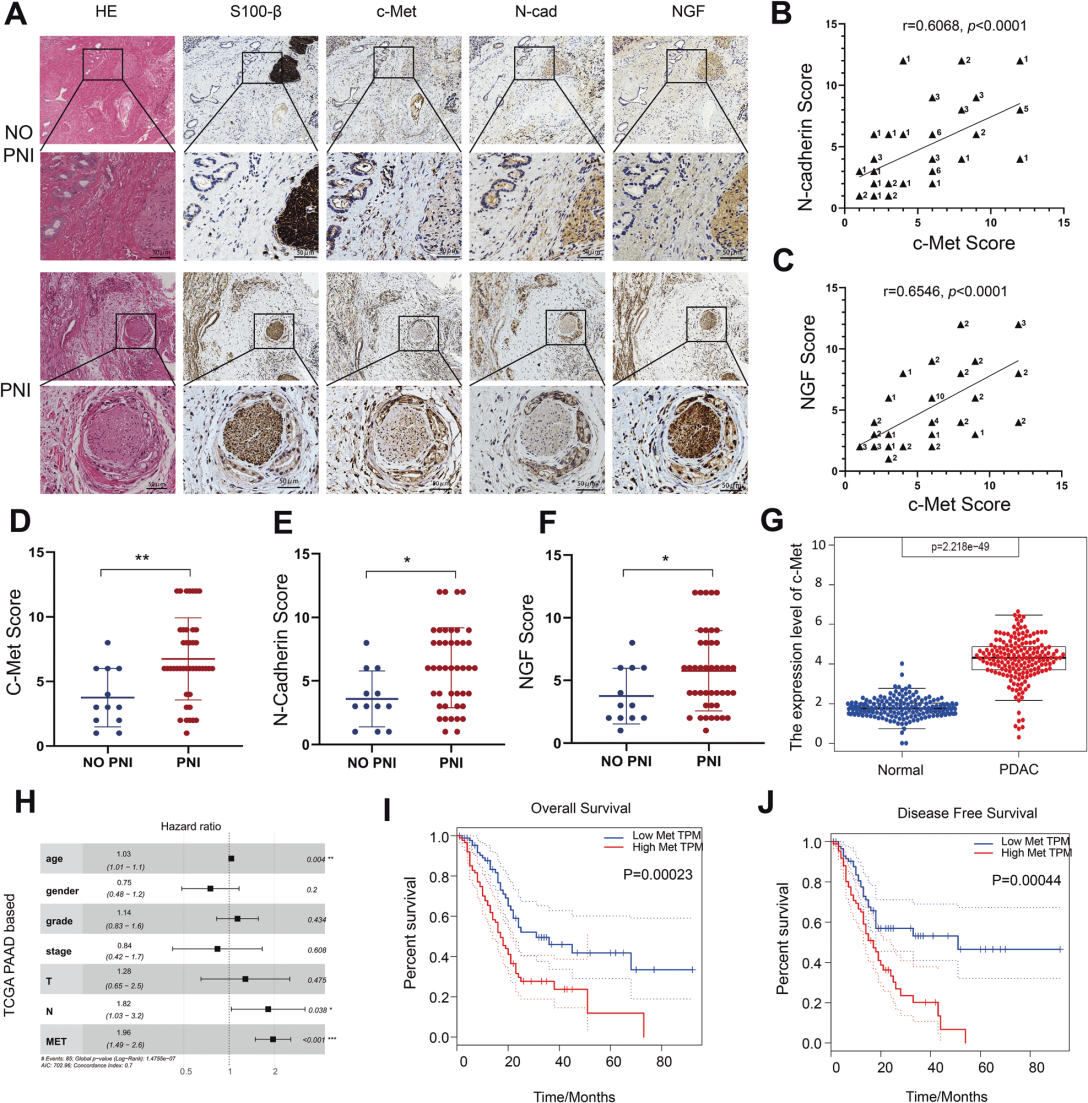
c-Met is overexpressed and associated with PNI in pancreatic cancer. **A** Representative histopathology (H&E staining) and IHC staining (S100β, c-Met, N-cadherin, and NGF) of pancreatic cancer tissue with or without PNI. **B** The positive correlation of c-Met expression with N-cadherin expression. The numbers of overlapping scores were labeled at the right of each point. **C** The positive correlation of c-Met expression with NGF expression. The numbers of overlapping scores were labeled at the right of each point. **D** The expression of c-Met in pancreatic cancer tissue with PNI and without PNI. **E** The expression of N-cadherin in pancreatic cancer tissue with PNI and without PNI. **F** The expression of NGF in pancreatic cancer tissue with PNI and without PNI. **G** Expression of c-Met in tumor tissues in the TCGA database to match TCGA normal and GTEx data. **H** The forest graph shows the HR and its 95% CI of the OS for c-Met in pancreatic cancer patients according to the TCGA database. **I** Overall survival of patients with pancreatic cancer and different levels of c-Met. **J** Disease-free survival of patients with pancreatic cancer and different levels of c-Met. *p < 0.05, **p < 0.01.

To analyze the relationship between c-Met and PNI in pancreatic cancer, we performed immunohistochemical staining to examine c-Met expression in human pancreatic cancer tissues with or without PNI. A nerve-specific marker, S100-β, was used to show the location of nerves. The c-Met level was higher in pancreatic cancer tissue with PNI than in pancreatic cancer tissues without PNI (Fig. 1A, D). Additionally, the expression level of N-cadherin was higher in pancreatic cancer tissue with PNI at the same location. We further confirmed NGF expression in human pancreatic cancer tissues by showing that NGF expression was also higher in tissues with PNI (Fig. 1A, E, F). The results showed that the expression of c-Met has a positive relationship with N-cadherin and NGF (Figs. 1B, C). Thus, we assumed that c-Met is positively correlated with PNI in pancreatic cancer. We used bioinformatics technology to analyze c-Met expression in tumor tissues in the TCGA database to match TCGA normal and GTEx data. c-Met was more highly expressed in tumor tissues (Fig. 1G). We also found that the c-Met level, age, and lymph node metastasis status were independent risk factors for OS in pancreatic cancer patients in TCGA datasets (Fig. 1H). Further analysis of the relationship between c-Met and the overall survival and disease-free survival of patients with pancreatic cancer was based on TCGA datasets on the GEPIA website [19]. The results showed high expression of c-Met may indicate a poor prognosis and high recurrence rate (Fig. 1I, J).

### Activating the HGF/c-Met signaling pathway promotes PNI in pancreatic cancer

**Fig. 2:**
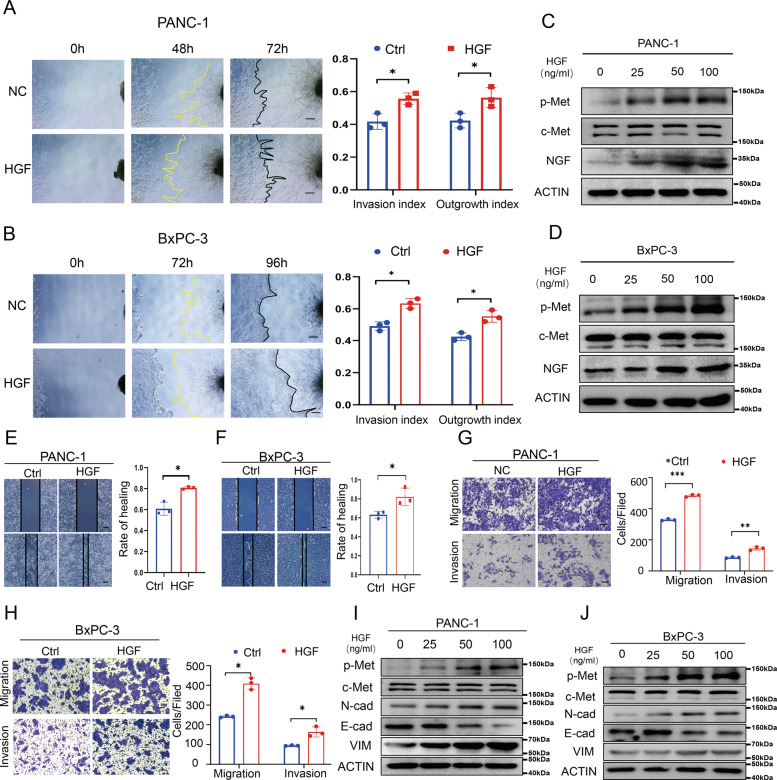
Activating the HGF/c-Met signaling pathway promotes PNI in pancreatic cancer. **A**, **B** Cancer cell-DRG coculture model for the control and HGF groups in PANC-1 cells and BxPC-3 cells. **C**, **D** Western blot analysis of the expression of p-Met, c-Met, NGF at different HGF concentrations in PANC-1 cells and BxPC-3 cells. **E**, **F** Wound healing assay of the control and HGF groups in PANC-1 cells and BxPC-3 cells. **G**, **H** Transwell-based assay of the control and HGF groups in PANC-1 cells and BxPC-3 cells. **I**, **J** Western blot analysis of the expression of p-Met, c-Met, N-cadherin, E-cadherin, and Vimentin in PANC-1 cells and BxPC-3 cells treated with different HGF concentrations. *p < 0.05, **p < 0.01, ***p < 0.001, ****p < 0.0001.

The crosstalk between cancer cells and nerves plays a vital role in PNI mediated by neurotrophins secreted by cancer cells. In addition, the mobility and invasion of cancer cells are critical, and the higher invasion and migration characteristics of pancreatic cancer contribute to PNI [9]. In the present study, to assess the biological significance of activating the HGF/c-Met signaling pathway in the crosstalk between nerves and cancer cells, a cancer cell-DRG (dorsal root ganglion) coculture system was established, and cancer migration toward DRG and neurite outgrowth was measured. Activating the HGF/c-Met signaling pathway increased the cancer cell invasion index and DRG outgrowth index (Fig. 2A, B). Furthermore, the expression of NGF was detected by western blotting revealed that activating the HGF/c-Met signaling pathway promotes NGF expression in the pancreatic cancer cell lines PANC-1 cells (Fig. 2C and Supplementary Fig. S1C) and BxPC-3 cells (Fig. 2D and Supplementary Fig. S1F). Furthermore, the wound healing assay revealed that activating c-Met by recombinant HGF promotes the healing abilities of pancreatic cancer cell lines (Fig. 2E, F). In addition, the result of immunofluorescence staining for wound healing assay after treatment with HGF showed that the expression of p-Met of BxPC-3 cells that migrated to the gap front was higher (Supplementary Fig. S2). The Transwell-based assay showed that activating the HGF/c-Met signaling pathway promotes the migration and invasion abilities of pancreatic cancer cell lines (Fig. 2G, H). Additionally, higher concentrations of HGF resulted in dose-dependent increases in the expression of N-cadherin and Vimentin and decreases in the expression of E-cadherin in PANC-1 cells (Fig. 2I and Supplementary Figs. S3A–E) and BxPC-3 cells (Fig. 2J and Supplementary Figs. S3F–J). PHA-665752 can effectively inhibit the phosphorylation level of c-Met in PANC-1 cells (Supplementary Figs. S4A and S4B) and BxPC-3 cells (Supplementary Figs. S4G and 4H) and N-cadherin, Vimentin and NGF were also effectively suppressed but E-cadherin expression was upregulated in PANC-1 cells (Supplementary Figs. S4A and S4C–F) and BxPC-3 cells (Supplementary Figs. S4G and 4I–L). Overall, these data showed that activating the HGF/c-Met signaling pathway facilitates PNI.

### Blocking the HGF/c-Met signaling pathway inhibits PNI in pancreatic cancer

**Fig. 3:**
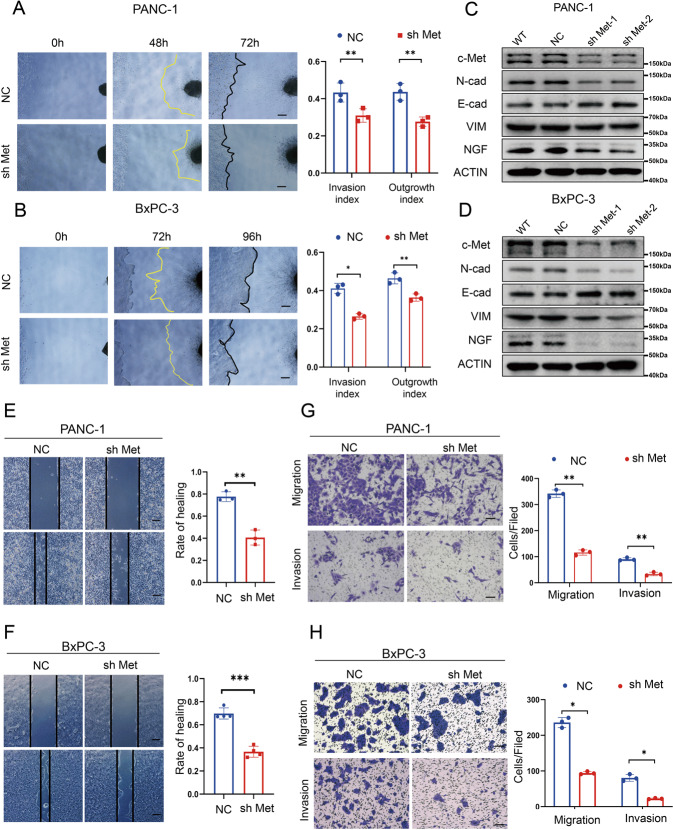
Blocking the HGF/c-Met signaling pathway inhibits PNI in pancreatic cancer. **A**, **B** Cancer cell-DRG coculture model for the NC and sh Met groups in PANC-1 cells and BxPC-3 cells. **C**, **D** Western blot analysis of the expression of c-Met, N-cadherin, E-cadherin, Vimentin, and NGF in the wild-type, NC, sh Met-1, and sh Met-2 groups in PANC-1 cells and BxPC-3 cells. **E**, **F** Wound healing assay of the NC and sh Met groups in PANC-1 cells and BxPC-3 cells. **G**, **H** Transwell-based assay of the NC and sh Met groups in PANC-1 cells and BxPC-3 cells. *p < 0.05, **p < 0.01, ***p < 0.001, ****p < 0.0001.

To further validate the c-Met-mediated finding in PNI, the lentiviral vector c-Met-shRNA was used to suppress c-Met expression in pancreatic cancer cell lines. The cancer cell-DRG coculture system showed that c-Met knockdown decreases the cancer cell invasion index and DRG outgrowth index (Fig. 3A, B). Western blotting also showed that suppressing c-Met expression downregulates the expression of NGF, N-cadherin, and Vimentin but upregulated the expression of E-cadherin (Fig. 3C, D and Supplementary Fig. S5A–J). Furthermore, the wound healing and Transwell-based assays showed that knockdown of c-Met expression impairs the migration and invasion abilities of the PANC-1 cell line (Fig. 3E, F) and BxPC-3 cell line (Fig. 3G, H). These results indicated that c-Met ablation inhibits PNI.

### Activating the HGF/c-Met pathway promotes PNI in pancreatic cancer by activating mTOR/NGF axis

**Fig. 4:**
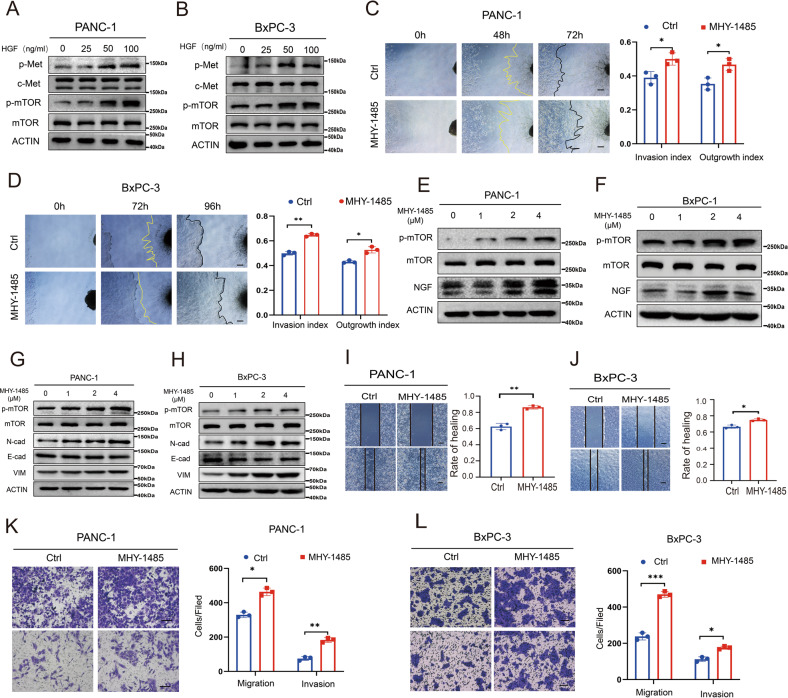
The HGF/c-Met pathway promotes PNI in pancreatic cancer by activating mTOR/NGF axis. **A**, **B** Western blot analysis of the expression of p-Met, c-Met, p-mTOR, and mTOR in PANC-1 cells and BxPC-3 cells treated with different concentrations of HGF. **C**, **D** Cancer cell-DRG coculture model for the control and MHY-1485 groups in PANC-1 cells and BxPC-3. **E**, **F** Western blot analysis of NGF expression in PANC-1 cells and BxPC-3 cells treated with different MHY-1485 concentrations. **G**, **H** Western blot analysis of the expression of p-mTOR, mTOR, N-cadherin, E-cadherin, and Vimentin in PANC-1 cells and BxPC-3 cells treated with different MHY-1485 concentrations. **I**, **J** Wound healing assay of the control and MHY-1485 groups in PANC-1 cells and BxPC-3 cells. **K**, **L** Transwell-based assay of the control and MHY-1485 groups in PANC-1 cells and BxPC-3 cells. *p < 0.05, **p < 0.01, ***p < 0.001.

Next, we investigated the underlying mechanisms by which the HGF/c-Met signaling pathway promotes PNI in pancreatic cancer. In our study, activating the HGF/c-Met signaling pathway increased the phosphorylation level of mTOR (Fig. 4A, B, Supplementary Figs. S6C and S6G). To assess the biological significance of activating mTOR in PNI of pancreatic cancer, MHY-1485 (mTOR activator that targets the ATP domain of mTOR) was used to treat pancreatic cancer cells. We investigated the crosstalk between pancreatic cancer cells and nerves after activating mTOR. The cancer cell-DRG coculture system demonstrated that activating mTOR increases the cancer cell invasion index and DRG axon growth index (Fig. 4C, D). Additionally, activating mTOR upregulated NGF expression in pancreatic cancer cell lines (Fig. 4E-F, Supplementary Figs. S7C and S7F). Western blotting showed that activating mTOR upregulated the expression of N-cadherin and Vimentin but downregulated the expression of E-cadherin (Fig. 4G–H, Supplementary Figs. S8A–J). The wound-healing assay showed that mTOR activation by MHY-1485 promotes the wound healing ability of pancreatic cancer (Fig. 4I, J). The result of immunofluorescence staining for wound healing assay after treatment with MHY-1485 showed that the expression of p-mTOR of BxPC-3 cells that migrated to the gap front was higher (Supplementary Fig. S9). The Transwell-based assay showed that the migration and invasion capabilities of cancer cells were increased by activating mTOR (Fig. 4K, L). In addition, PF-04691502 (an inhibitor that can inhibit mTOR phosphorylation on 2448) can inhibit the activation of mTOR which was upregulated by HGF. In addition, the use of mTOR inhibitors can inhibit EMT and the expression of NGF which were promoted by the activation of HGF/c-Met signaling pathway (Supplementary Figs. S10A–L). These data indicated that the HGF/c-Met pathway promotes PNI in pancreatic cancer by activating mTOR/NGF axis.

### Blocking the HGF/c-Met/mTOR signaling pathway by knocking down the expression of mTOR inhibits PNI of pancreatic cancer

**Fig. 5:**
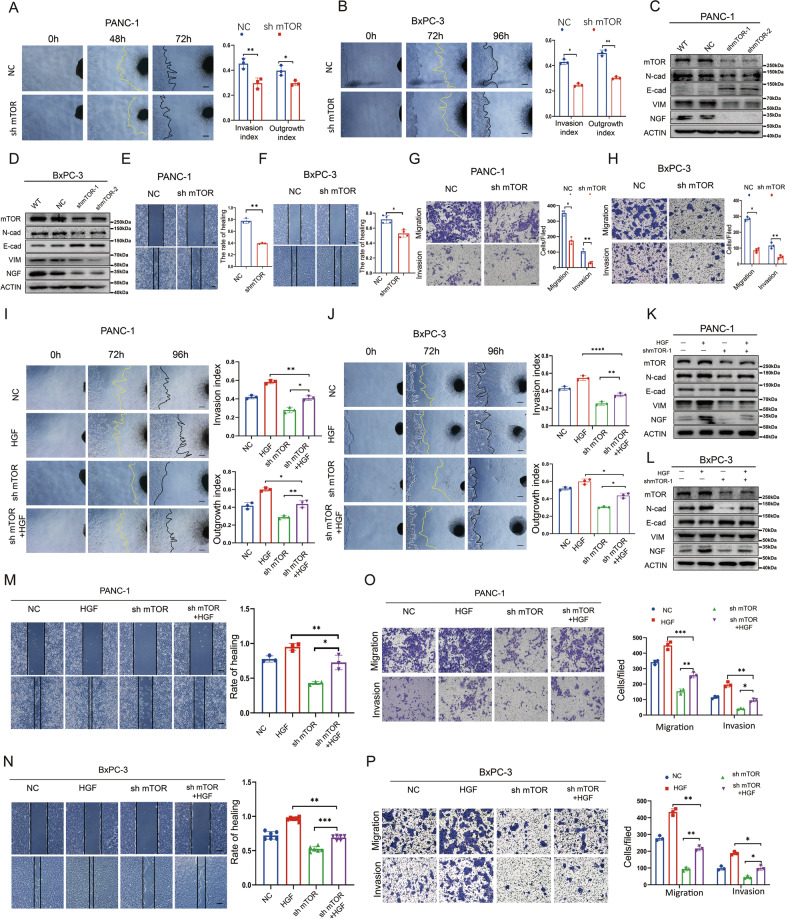
Blocking the HGF/c-Met/mTOR signaling pathway by knocking down the expression of mTOR inhibit PNI of pancreatic cancer. **A**, **B** Cancer cell-DRG coculture model for the NC and sh mTOR groups in PANC-1 and BxPC-3 cells; **C**, **D** Western blot analysis of the expression of mTOR, N-cadherin, E-cadherin, Vimentin, and NGF in the wild-type, NC, sh mTOR-1, and sh mTOR-2 groups in PANC-1 cells and BxPC-3 cells. **E**, **F** Wound healing assay of the NC and sh mTOR groups in PANC-1 cells and BxPC-3 cells. **G**, **H** Transwell-based assay of the NC and sh mTOR groups in PANC-1 cells and BxPC-3 cells. **I**, **J** Cancer cell-DRG coculture model for the NC, HGF, sh mTOR, and sh mTOR+HGF groups in PANC-1 cells and BxPC-3 cells. **K**, **L** Western blot analysis of the expression of mTOR, N-cadherin, E-cadherin, Vimentin, and NGF in the NC, HGF, sh mTOR, and sh mTOR+HGF groups in PANC-1 cells and BxPC-3 cells. **M**, **N** Wound healing assay of the NC, HGF, sh mTOR, and sh mTOR+HGF groups in PANC-1 cells and BxPC-3 cells. **O**, **P** Transwell-based assay of the NC, HGF, sh mTOR, and sh mTOR+HGF groups in PANC-1 cells and BxPC-3 cells. *p < 0.05, **p < 0.01, ***p < 0.001.

To further understand the role of mTOR in the progression of the HGF/c-Met pathway promoting PNI in pancreatic cancer, the lentiviral vector mTOR-shRNA was used to suppress mTOR expression in pancreatic cancer cell lines. As observed in the cancer cell-DRG coculture system, mTOR knockdown in cancer cells decreased the cancer cell invasion index and DRG axon growth index (Fig. 5A, B). Western blotting demonstrated that suppressing mTOR expression downregulates NGF expression. Furthermore, western blotting revealed that suppressing mTOR expression inhibits the progression of EMT in pancreatic cancer by downregulating the expression of N-cadherin and Vimentin but upregulating the expression of E-cadherin (Fig. 5C, D and Supplementary Figs. S11A–J). In addition, the wound healing and Transwell-based assays showed that decreasing mTOR expression impairs the migration and invasion abilities of PANC-1 cells (Fig. 5E, G) and BxPC-3 cells (Fig. 5F, H).

Given that suppressing mTOR expression inhibits PNI in pancreatic cancer, we speculated whether mTOR is required for the HGF/c-Met signaling pathway to exert its promoting effects on PNI in pancreatic cancer. To verify our hypothesis, pancreatic cancer cells (sh NC and sh mTOR) were treated with recombinant HGF (100 ng/mL). In the cancer cell-DRG coculture system, the cancer cell invasion index and DRG axon growth index, which were inhibited by knockdown of mTOR expression, were recovered by HGF (Fig. 5I, J). The cancer cell invasion index and DRG axon growth index were suppressed in the HGF + sh mTOR group compared with the HGF group (Fig. 5I, J). Additionally, compared with the HGF group, the expression of N-cadherin, Vimentin, and NGF was downregulated, but E-cadherin was upregulated in the HGF + sh mTOR group (Fig. 5K–L and Supplementary Figs. S12A–J). We also found that compared with the sh mTOR group, activating the HGF/c-Met signaling pathway recovered the N-cadherin and Vimentin expression of pancreatic cancer cells but decreased the E-cadherin expression in the HGF + sh mTOR group (Fig. 5K–L and Supplementary Fig. S12A–J). More importantly, NGF expression was also recovered (Fig. 5K–L and Supplementary Figs. S12A–J). The wound healing and Transwell-based assays showed that, compared with the sh mTOR group, HGF recovered the migration and invasion abilities of pancreatic cancer cells in the HGF + sh mTOR group (Fig. 5M–P). Compared with the HGF group, invasion and migration were blocked in the HGF + sh mTOR group (Fig. 5M–P). We revealed that mTOR is required for the HGF/c-Met signaling pathway to exert its promoting effects on PNI in pancreatic cancer.

### Blocking the HGF/c-Met signaling pathway suppresses PNI in vivo

**Fig. 6:**
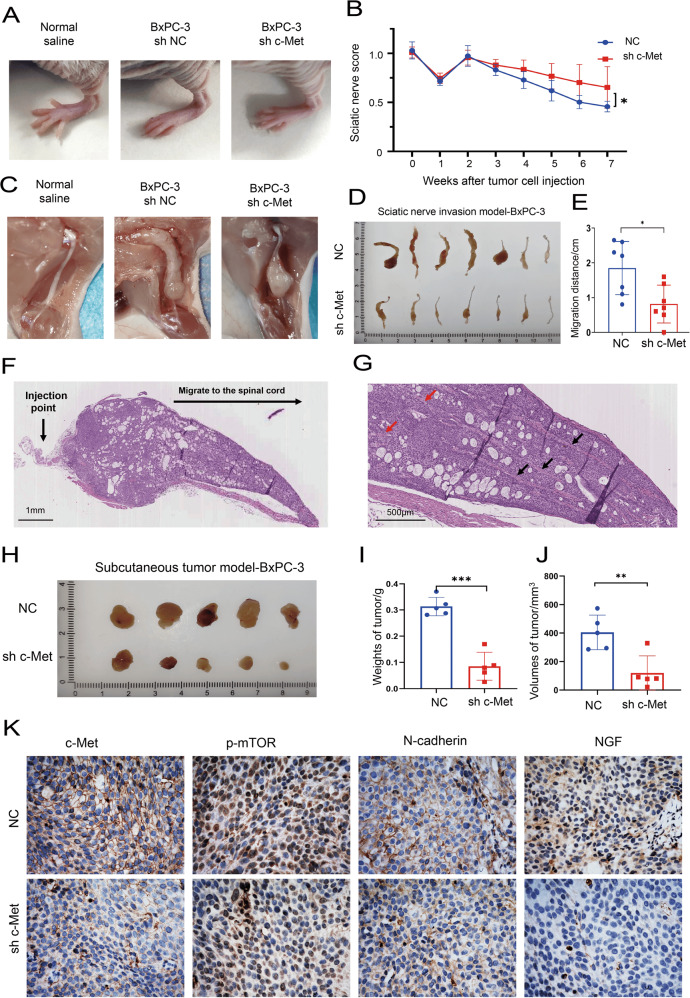
Knockdown of c-Met expression suppresses PNI in vivo. **A** Limb function of nude mice in the normal saline, NC, and sh Met groups. **B** Sciatic nerve score of nude mice in the NC and sh Met groups. **C** Anatomy results of the growth and migration of cancer cells along the sciatic nerve. **D**, **E** Distance of cancer cells migrating along the sciatic nerve. **F**, **G** Cancer cells invade and migrate along the sciatic nerve to the spinal cord. The neural structure is destroyed during cell invasion (red arrow). Nervous tissue continuity in the invasion front is better (black arrow). **H** Macroscopic images of the subcutaneous tumor model result from the NC and sh Met groups. **I** Weights of the tumors in the NC and sh Met groups. **J** Volumes of the tumors in the NC and sh Met groups. **K** The expression of c-Met, p-mTOR, N-cadherin, and NGF was detected by IHC in the NC and sh Met group. *p < 0.05, **p < 0.01, ***p < 0.001.

To further understand the crosstalk between pancreatic cancer cells and nerves and whether blocking the HGF/c-Met signaling pathway inhibits PNI in pancreatic cancer in vivo, a sciatic nerve invasion model was established by injecting BxPC-3 cells (sh NC vs. sh c-Met). Cancer cells injected into the sciatic nerve severely damaged the limb function of nude mice. When the expression of c-Met was knocked down, limb function damage was reduced (Fig. 6A). The sciatic nerve score was measured every week in this study. As shown by the sciatic nerve score curves, the sciatic nerve score of the sh c-Met group was higher than that of the sh NC group (Fig. 6B). Anatomy results also showed that suppressing c-Met expression inhibits the growth and migration of cancer cells along the sciatic nerve (Fig. 6C). In the sciatic nerve invasion model, cancer cells can migrate along the sciatic nerve, and the distance of cancer migration along the sciatic nerve can indicate the PNI ability of cancer cells. Measuring the distance of cancer cells migrating along the sciatic nerve, we found that inhibiting the expression of c-Met can significantly suppress cancer cell migration along the sciatic nerve (Fig. 6D, E). In addition, cancer cells that invade the sciatic nerve migrate along the nerve to the spinal cord (Fig. 6F). There is enlargement of nerves in the process of cancer cells invading and migrating along the nerve and the nerve structure is destroyed by the cancer cells. Inhibiting the expression of c-Met of cancer cells can reduce the damage of tumor cells to nerves (Fig. 6G and Supplementary Fig. S13). The results also showed that cancer cells with high expression of c-Met have a richer blood supply in the sciatic nerve (Supplementary Fig. S13).

Increased cancer cell viability and enhanced invasion and migration ability can facilitate cancer cells invading nerves. NGF secreted by cancer cells can promote nerve fiber regeneration and recruit nerves. A subcutaneous pancreatic cancer xenograft model was established in nude mice to detect the viability, EMT, and NGF expression of BxPC-3 cells (sh NC vs. sh c-Met). Knocking down c-Met significantly inhibited the weights and volumes of tumors (Fig. 6H–J). Additionally, we performed immunostaining and results revealed that N-cadherin expression is lower and weaker in the sh c-Met group than in the sh NC group, and p-mTOR and NGF expression are lower and weaker in the sh c-Met group than in the sh NC group (Fig. 6K and Supplementary Figs. S14A–D). These results indicated that downregulation of c-Met inhibits PNI in pancreatic cancer in vivo.

## Discussion

PNI is a complicated pathological phenomenon and a continuous and multistep process in which nerve cells, cancer cells, other supporting cells, and the microenvironment of tumors have changed [14]. The interaction, chemotactic movement, contact, and adhesion of nerve cells and cancer cells are crucial processes in PNI [14]. Cancer cells recruit nerves and promote nerve growth by secreting neurotrophic factors, which facilitate PNI in pancreatic cancer [14]. Furthermore, the enhanced viability, mobility, and invasion capacity of cancer cells are conducive to cancer cell invasion of nerve structure and contribute to the PNI [9]. Considering the important role of the above aspects in PNI, it is feasible to explore the mechanism of PNI by studying the interaction between cancer cells and nerves, the expression of neurotrophic factors, and the invasion and migration ability of cancer cells in the process of PNI.

Various neurotrophic factors are involved in the interaction between nerves and pancreatic cancer cells [14]. One of the most important neurotrophic factors is NGF. In genetically engineered mice harboring spontaneous pancreatic cancer, the suppression of NGF signaling slows the development of pancreatic disease [20]. Cancer cells secrete NGF through paracrine pathways to promote neuroplasticity, thereby recruiting nerves and facilitating cancer cell invasion of nerves. Anti-NGF inhibits the migration of pancreatic cells along the nerve to the thoracic spinal cord [20]. In another study, conditional pancreatic Ngf overexpression in KC (LSL-Kras^+/LSL-G12D^; Pdx1-Cre) mice increased TH^+^ sympathetic neurons, with prominent nerves and intrapancreatic ganglia. *Ngf* overexpression led to prominent PNI and enlarged intratumoral nerves [21]. Blocking of the NGF/Trk pathway inhibits proliferation and innervation and increases overall survival in KPC mice [21]. NGF plays an important role in the PNI and exploring the mechanism that promoting the expression of NGF is essential to understand the mechanism of PNI. In our study, activating the HGF/c-Met signaling pathway upregulated NGF expression via activating mTOR. Furthermore, activating the HGF/c-Met/mTOR signaling pathway in pancreatic cancer promotes the growth of DRG axons in the cancer cells-DRG coculture model. Commonly, NGF is an upstream regulator of the mTOR pathway and few studies have shown that mTOR can regulate the expression of NGF in cancer cells [22–24]. In the present study, we revealed that mTOR can regulate NGF expression as an upstream regulator in pancreatic cancer. However, the mechanism by which mTOR regulates NGF expression remains unclear. Multiple studies have shown that mTOR regulates the biological functions of cells by regulating downstream transcription factors such as HIF1α、MAF1、SERBPs [25]. Whether activating HGF/c-Met/mTOR signaling pathway directly regulates NGF expression by affecting downstream transcription factors remains unclear. In the following research, the mechanism of which transcription factors regulate NGF expression in the HGF/c-Met/mTOR signaling pathway will become the focus of our research.

PNI is considered to be the fourth route of cancer spread in addition to three common transfer routes (direction invasion, lymphatic spread, and hematogenic spread) [26]. Pancreatic cancer cells invading and migrating along nerves promote distant metastasis [9]. Increased invasion and migration ability contribute to cancer cells invading nerves. In addition, cancer cells with strong invasion and migration ability can migrate faster and farther along the nerve after invading nerves. In EMT progression, the migration and invasion capabilities of cancer cells increase [27]. Previous researchers found that activating mTOR promoted the invasion and metastasis of cancer [28, 29]. In addition, NGF also can promote the invasion and migration of liver cancer cells, prostate cancer cells, and pancreatic cancer cells [30–32]. In our study, activating HGF/c-Met/mTOR signaling pathway can promote the expression of NGF. The activation of the mTOR signaling pathway and the autocrine of NGF may together promote the invasion and migration ability of pancreatic cancer cells. Furthermore, blocking the HGF/c-Met/mTOR signaling pathway by knocking down the expression of mTOR inhibits the invasion and migration of pancreatic cancer cells. With the help of the sciatic nerve invasion model, we found that knocking down c-Met expression can significantly inhibit cancer cell migration along the sciatic nerve. Thus, blocking the HGF/c-Met/mTOR signaling pathway can inhibit cancer cells invading and migrating along nerves and may be a therapeutic target for PNI in pancreatic cancer.

Many patients with pancreatic cancer have abdominal pain or back pain during the development of the disease [33]. Severe and enduring pain influences the quality of life for pancreatic cancer patients and is related to the poor prognosis [34]. Pancreatic cancer pain is caused by many reasons and PNI contributes to the generation of cancer-associated pain. Tumor cells can destroy the nerve sheath in the process of PNI, which makes the nerve more sensitive to stimulation [35]. Vascularization of the tumor and the growth of new nerve fibers also contribute to pain generation [35, 36]. In addition, NGF plays an important role in pancreatic pain by modulating the expression and activity of TRPV1 [37]. In the present study, activating the HGF/c-Met signaling pathway can increase the expression of NGF. Blocking the HGF/c-Met signaling pathway can inhibit the damage of tumor cells to nerves and the vascularization of the tumor in nerves. These results suggest blocking the HGF/c-Met/mTOR signaling pathway may be a potential strategy for treating pancreatic cancer pain.

**Fig. 7:**
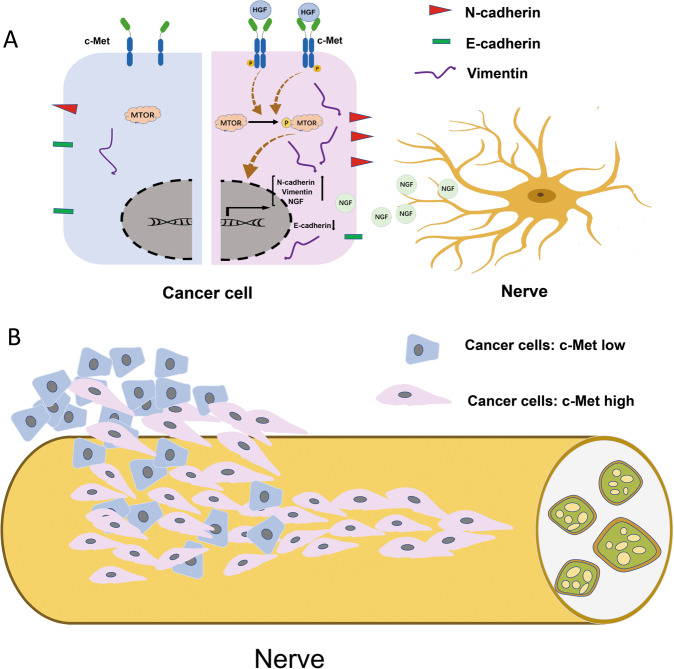
HGF/c-Met pathway facilitates the perineural invasion of pancreatic cancer by activating the mTOR/NGF axis. **A** Activating the HGF/c-Met signaling pathway upregulated NGF expression via activating mTOR. Pancreatic cancer cells can recruit nerves and facilitate cancer cell invasion of nerves by increasing the expression of NGF. On the other hand, activating the HGF/c-Met/mTOR signaling can increase the invasion and migration ability of pancreatic cancer cells by upregulating N-cadherin and Vimentin but downregulating E-cadherin. It will facilitate cancer cells invading nerves. **B** Pancreatic cancer cells with high expression of c-Met can invade nerves more easily and metastasize farther along the nerve. Blocking the HGF/c-Met signaling pathway suppresses cancer cell invasion nerves and metastasis along nerves to distant places.

In conclusion, our study has provided evidence supporting that the HGF/c-Met signaling pathway promotes PNI in pancreatic cancer by activating the mTOR/NGF axis and increasing the invasion and migration ability of pancreatic cancer cells (Fig. 7). These results deepen our understanding of the crosstalk between cancer cells and nerves in pancreatic cancer. Blocking the HGF/c-Met/mTOR signaling pathway may be an effective strategy for inhibiting PNI.

## Methods

### Reagents

The antibodies used in the study were as follows: anti-S100β (Cat No. 15146-1-AP, Proteintech, Wuhan, China), anti-N-cadherin (Cat No. 22018-1-AP, Proteintech, Wuhan, China), anti-E-cadherin (Cat No. 60335-1-Ig, Proteintech, Wuhan, China), anti-Vimentin (Cat No. 60330-1-Ig, Proteintech, Wuhan, China), anti-NGF (Cat No. ab52918, Abcam, Cambridge, UK), anti-c-Met (Cat No. ab51067, Abcam, Cambridge, UK), anti-p-c-Met (Cat No. 3077, CST, Danvers, MA, USA), anti-mTOR (Cat No. 2983S, CST, Danvers, MA, USA), anti-p-mTOR (Cat No. 5536S, CST, Danvers, MA, USA; Santa Cruz, CA, USA), anti-ACTIN (Cat No. 66009-1-Ig, Proteintech, Wuhan, China), HRP-conjugated secondary antibodies (Cat No. SA00001-1 and SA00001-2, Proteintech, Wuhan, China), and fluorescence-conjugated secondary antibody (Cat No. SA00013-1, SA00013-2, SA00013-3, and SA00013-4, Proteintech, Wuhan, China). Recombinant human HGF was purchased from Sino Biological (Beijing, China). MHY-1485 (mTOR agonist), PHA-665752 (c-Met inhibitor), and PF-04691502 (mTOR inhibitor) were purchased from MedChem Express (Monmouth Junction, NJ, USA).

### Human tissue samples

We obtained pancreatic cancer samples (55 cases) from the Department of Hepatobiliary Surgery, the First Affiliated Hospital of Xi’an Jiaotong University between 2017 and 2019 after receiving approval from the Ethical Committee at the First Affiliated Hospital of Xi’an Jiaotong University. And consent was obtained from all subjects. The pathological PNI status was assessed according to the definition proposed by Liebig [8]: cancer cell in close proximity to nerve and involving at least 33% of its circumference or tumor cells within any of the 3 layers of the nerve sheath. The pathological factors were examined by two pathologists. The clinical pathological data are summarized in Supplementary Table 1.

### Immunohistochemistry (IHC)

Immunohistochemistry was performed as described previously. Briefly, tissue sections were incubated with primary antibodies at a suitable concentration at 4 °C overnight. After washing three times with PBS, the appropriate biotinylated secondary antibodies were added to the sections, which were incubated with secondary antibodies for 1 h at room temperature. After washing three times with PBS, the sections were visualized with DAB, and hematoxylin was used for counterstaining. Based on the staining intensity, the samples were scored as follows: light-yellow (1), brown-yellow (2), brown (3). Based on the percentage of positive cells, the sample were scored as follows: <25% (1), ≥25% and <50% (2), ≥50% and <75% (3), ≥25% (4). The final score was defined as intensity score multiplied by percentage score. The final score which was less than 5 was defined as low expression and score more than 5 was defined as high expression.

### Cell lines

Human pancreatic cancer cell lines (PANC-1 and BxPC-3) were purchased from the Chinese Academy of Sciences Cell Bank of Type Culture Collection (Shanghai, China). Cell lines were recently authenticated by STR profiling and tested for mycoplasma contamination. The PANC-1 cell line was cultured in DMEM (Gibco, Grand Island, NY, USA), and the BxPC-3 cell line was cultured in 1640 medium (Gibco, Grand Island, NY, USA) in an incubator with 5% CO2 at 37 °C. All media were supplemented with 10% fetal bovine serum and 1% penicillin-streptomycin.

### Stable shRNA lentiviral transfection

c-Met shRNA (sh c-Met), mTOR shRNA (sh mTOR), and negative control shRNA (sh NC) were cloned into GV248 lentiviral vectors, which are powerful tools for modifying the genes of eukaryotic cells. Lentivirus was used to transfect pancreatic cancer cells and downregulate c-Met and mTOR expression. All shRNAs were purchased from GeneChem Co, Ltd. (Shanghai, China). Cells were seeded in 24-well plates at 1 × 10^4^ cells/well, and 5 μL of lentiviral particles were added to the plates. The cell culture medium was changed to fresh complete medium after 8 h. Puromycin was used to select the cells transfected successfully with virus particles. After 3 weeks, puromycin-resistant cells were isolated for subsequent study.

### Immunofluorescence staining

All cells on the sections were fixed with 4% paraformaldehyde, followed by washing three times in PBS and blocking with 5% BSA. The sections were incubated with primary antibodies at a suitable concentration at 4 °C overnight. After washing three times with PBS, fluorescence-conjugated secondary antibodies were added to the sections, which were incubated with secondary antibodies for 1 h at room temperature. DAPI was used to stain nuclei for 1 min. A Zeiss Instruments microscope was used to acquire images.

### Wound healing assay

The wound healing assay was performed to assess the migration ability of cancer cells. Briefly, pancreatic cancer cells were seeded in 6-well plates at an appropriate confluence. A 200-μL pipette tip was used to create a wound, and dead cells were removed with PBS. All cancer cells were cultured with serum-free medium, and images were acquired at suitable time points. ImageJ software (National Institutes of Health, Bethesda, MD, USA) was used to evaluate the migration ability of cancer cells.

### Transwell-based assay

The Transwell-based assay was used to assess the migration and invasion abilities of pancreatic cancer cells. When we assessed the migration ability of pancreatic cancer cells, the pancreatic cancer cells were suspended in serum-free medium, and 1 × 10^4^ cells were seeded in the upper wall of the chamber. When we assessed the invasion ability of pancreatic cancer cells, the upper face of the chamber was coated with Matrigel, and 2 × 10^4^ cells were seeded in the upper wall of the chamber. Complete medium was added to the lower chamber. After culturing for 48 h, pancreatic cancer cells in the upper wall were removed, and the pancreatic cancer cells on the lower surface were fixed with 4% paraformaldehyde and stained with 1% crystal violet. Images were obtained using a microscope, and the number of cells in each field was recorded.

### Western blotting

Pancreatic cancer cells were lysed and the total protein was extracted with RIPA buffer (Beyotime, Guangzhou, China). After SDS-PAGE, the total protein was transferred to PVDF membranes (Roche, Penzberg, Germany). Next, the membranes were saturated with a rapid blocking buffer (NCM Biotech, Suzhou, China) for 20 min. After washing three times with PBST, the PVDF membranes were incubated with the primary antibody overnight at 4 °C. After washing three times with PBST again, the membranes were incubated with the secondary antibody for 1 h at room temperature. Protein expression was detected using an enhanced chemiluminescence (ECL) kit (NCM Biotech, Suzhou, China) and a ChemiDoc XRS System (Bio-Rad, CA, USA). ImageJ software (National Institutes of Health, Bethesda, MD, USA) was used to evaluate the densitometric of the bands and the results were normalized to the ACTIN level.

### Cancer cell-DRG coculture system

The cancer cell-DRG coculture system was performed according to the method described previously [13]. Briefly, a drop of cell suspension was seeded in a 24-well plate. After euthanizing the newborn rat, the dorsal root ganglia (DRG) were obtained from the intervertebral foramen. The immunofluorescence method is used to identify ganglia and axons with S100β, NF200, TUJ1 (Supplementary Fig. S15). The axons around the DRG were removed, and the DRG were seeded 2 mm from the edge of the cell drop. Matrigel was then added to cover the cancer cells and DRG. Cancer cells and DRG can communicate with each other by the bridge formed by Matrigel. After incubation for 1 h, a complete medium was added to the 24-well plates. The cancer cell-DRG coculture system was established, and images were acquired every day. The growth of cancer cells and DRG was recorded. The invasion index was used to assess cancer cell migration and invasion (invasion index = α/γ), and the growth index was used to evaluate the ability of dorsal root ganglion axons to grow toward cancer cells (outgrowth index = β/γ) (Supplementary Fig. S16).

### In vivo model

The subcutaneous pancreatic cancer xenograft model was used to evaluate the viability and epithelial-mesenchymal transition (EMT) of cancer cells. BALB/c nude mice were randomized into two groups and the number of each group was 5 and the sample size was estimated from preliminary experiments or reports in the literature. Pancreatic cancer cells (BxPC-3 sh NC and BxPC-3 sh c-Met) were suspended in serum-free medium at 1 × 10^7^/mL, and 100 μL was injected subcutaneously into six-week-old male nude mice (*n* = 5). After 8 weeks, the nude mice were sacrificed, and the subcutaneous tumors were isolated and weighed, the volume was calculated by 1/2 × length × width^2^. The tumors were fixed in formalin as quickly as possible.

The sciatic nerve invasion model was used to evaluate the crosstalk between cancer cells and nerves in vivo. Cancer cells were injected into nude mouse sciatic nerves to simulate the process of cancer cells invading into and migrating along nerves. BALB/c nude mice were randomized into two groups and the number of each group was 7 and the sample size was estimated from preliminary experiments or from reports in the literature. Pancreatic cancer cells (BxPC-3 sh NC and BxPC-3 sh c-Met) were suspended in Matrigel at 1 × 10^7^/mL, and 2 μL was injected into the sciatic nerves of six-week-old male nude mice. The sciatic nerve score was recorded every week. The extension length between the first and fifth toes of the hind limbs was calculated as the sciatic nerve score. After 7 weeks, the nude mice were sacrificed, and the sciatic nerves were isolated. The distance of cancer cells migrating along nerves was calculated. All experimental protocols were approved by the Ethical Committee of Xi’an Jiaotong University.

### Statistical analysis

Statistical analysis was performed using SPSS (version 15.0; SPSS, Chicago, IL, USA). The results were expressed as mean ± SD, and minimum three biological replicates were used. A two-tailed paired Student’s *t* test was used to evaluate the difference between the two groups of in vitro experiments. A two-tailed unpaired Student’s *t* test was used to evaluate the difference between the two groups of in vivo experiments. And differences between multiple groups were assessed by one-way ANOVA. All data are tested for normality and homogeneity of variances before differences analysis. *P* < 0.05 was considered statistically significant.

## Supplementary information

checklist

Supplementary

## Data Availability

The original contributions presented in the study are included in the article/supplementary material. Further inquiries can be directed to the corresponding authors.
