# Supplementary material for: HGF/c-Met pathway facilitates the perineural invasion of pancreatic cancer by activating the mTOR/NGF axis
Source: Cell Death Dis. 2022 Apr 21;13(4):387. doi: 10.1038/s41419-022-04799-5 (PMC9023560; doi:10.1038/s41419-022-04799-5)
Supplement: Supplementary file 2 — Supplementary [file 41419_2022_4799_MOESM2_ESM.docx]

Table 1 Baseline Data Sheet of Patients with pancreatic cancer

| Characteristic | Low expression of c-Met | High expression of c-Met | p |
| --- | --- | --- | --- |
| n | 18 | 37 |  |
| Age |  |  | 0.786 |
| >55 | 12 | 26 |  |
| ≤55 | 6 | 11 |  |
| Gender |  |  | 0.291 |
| Femal | 11 | 17 |  |
| Male | 7 | 20 |  |
| T Stage |  |  | 0.517 |
| T1 | 5 | 6 |  |
| T2 | 6 | 12 |  |
| T3 | 2 | 10 |  |
| T4 | 5 | 9 |  |
| N Stage |  |  | 0.374 |
| N0 | 12 | 20 |  |
| N1&N2 | 6 | 17 |  |
| Perineural invasion |  |  | 0.005 |
| Yes | 10 | 33 |  |
| No | 8 | 4 |  |

**Figure S1**


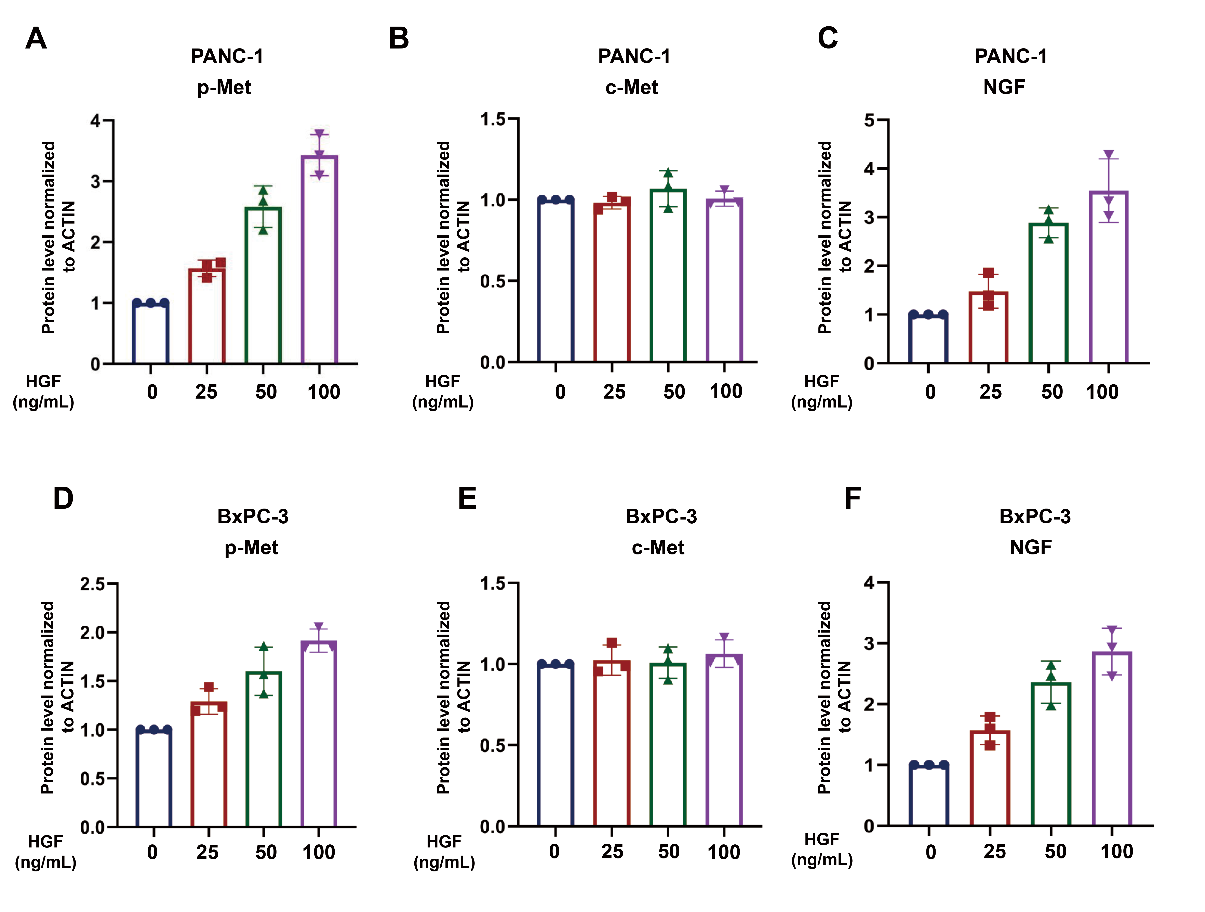


**Figure S1** (A-C) The relative protein level of p-Met (A), c-Met (B) and NGF (C) normalized to the ACTIN after treatment with HGF in PANC-1 cells; (D-F) The relative protein level of p-Met (D), c-Met (E) and NGF (F) normalized to the ACTIN after treatment with HGF in BxPC-3 cells.

**Figure S2**


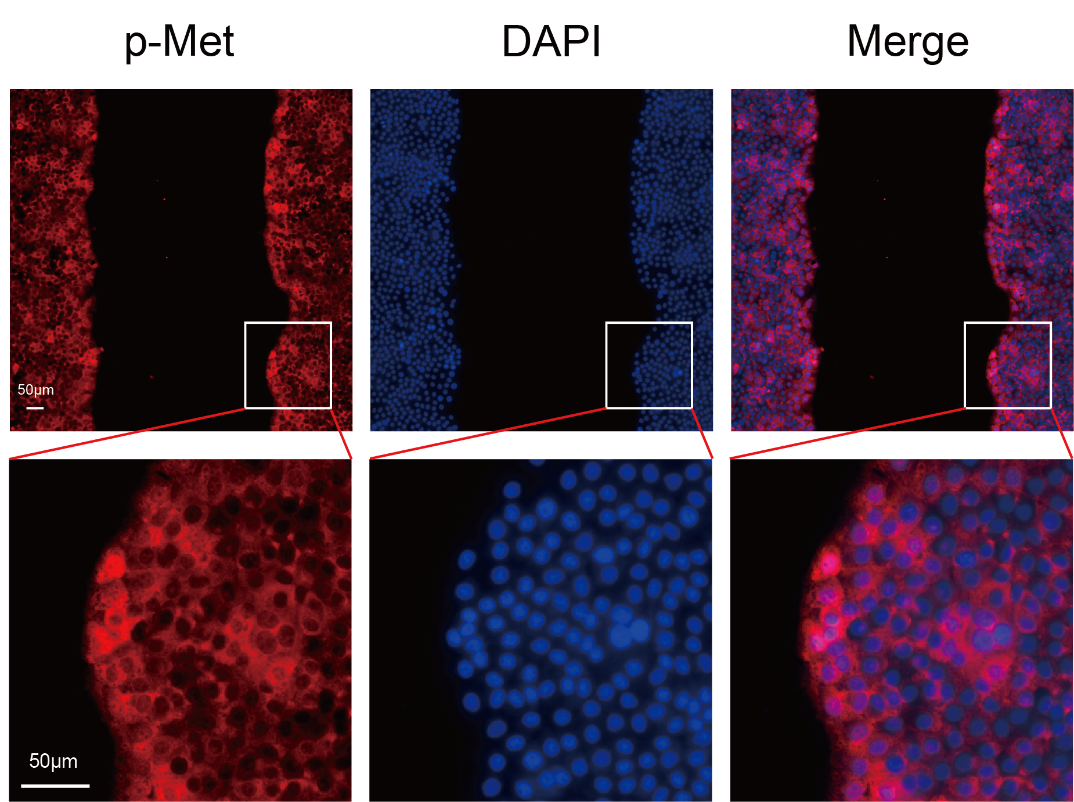


**Figure S2** Immunofluorescence staining for wound healing assay after treatment with HGF shows that the expression of p-Met of BxPC-3 cells that migrated to the gap front is higher.

**Figure S3**


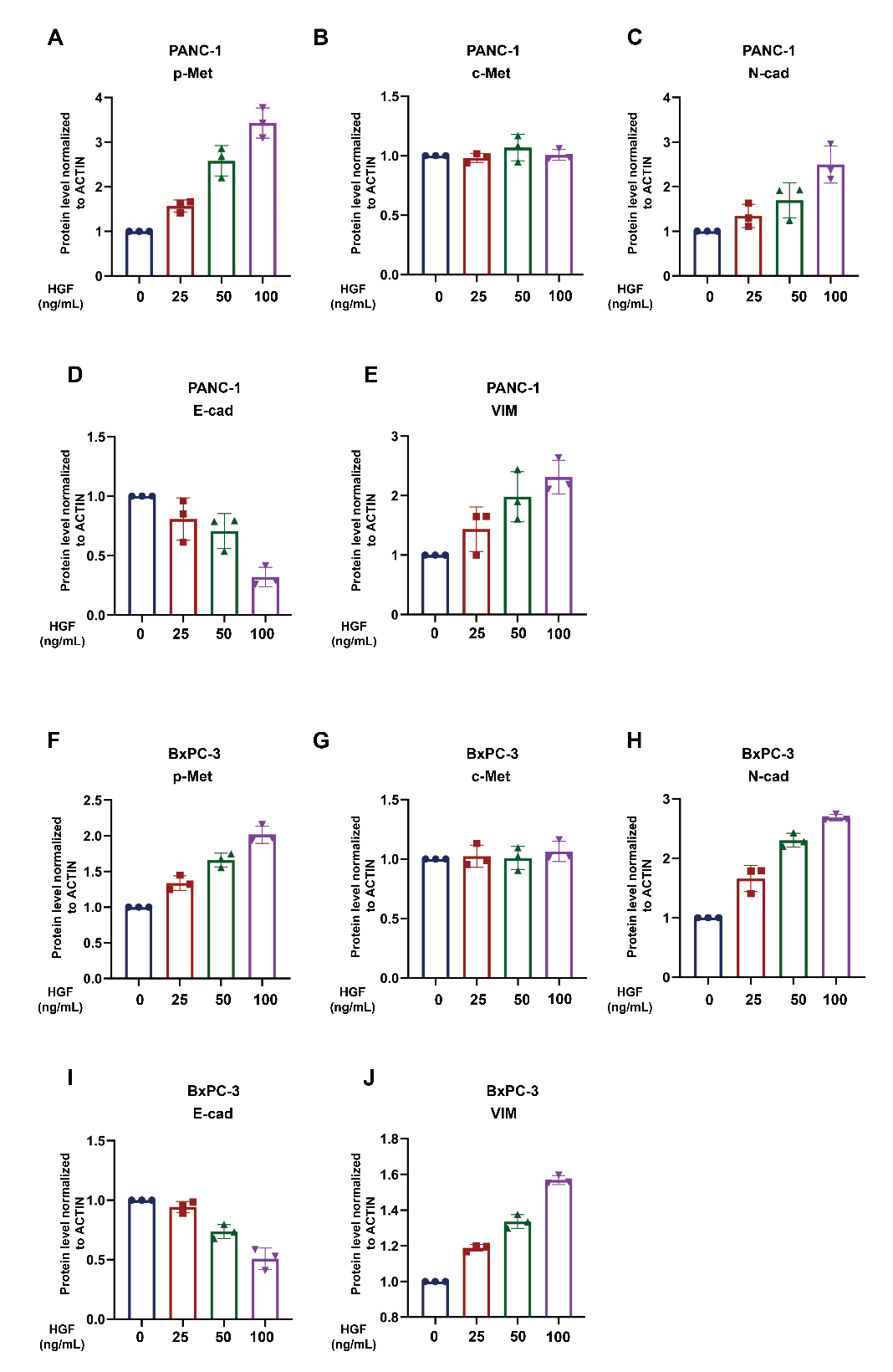


**Figure S3** (A-E) The relative protein level of p-Met (A), c-Met (B), N-cadherin (C), E-cadherin (D) and Vimentin (E) normalized to the ACTIN after treatment with HGF in PANC-1 cells; The relative protein level of p-Met (F), c-Met (G), N-cadherin (H), E-cadherin (I) and Vimentin (J) normalized to the ACTIN after treatment with HGF in BxPC-3 cells.

**Figure S4**


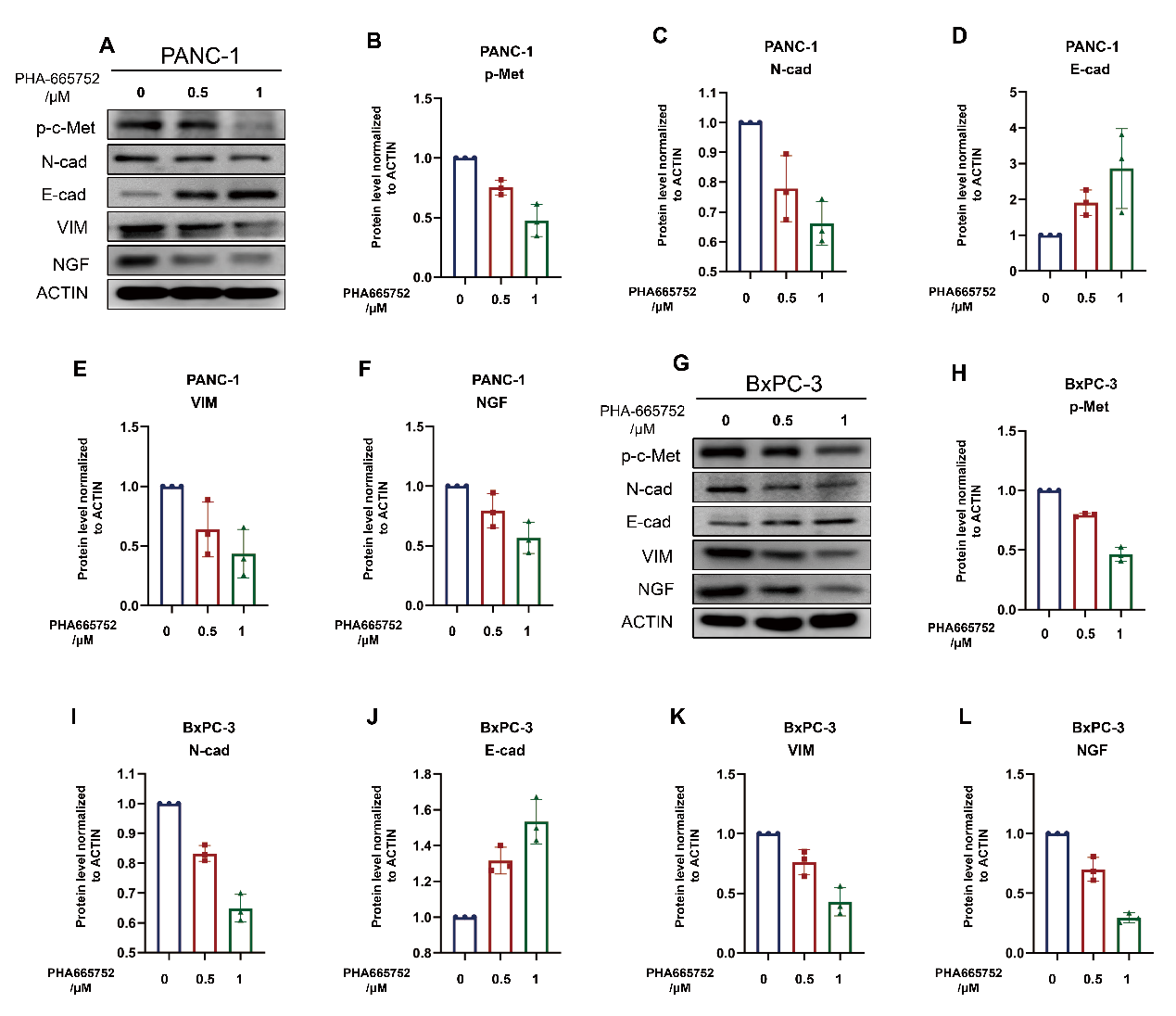


**Figure S4** (A) Western blot analysis of the expression of p-Met, N-cadherin, E-cadherin, Vimentin and NGF at different PHA-665752 concentrations in PANC-1 cells；(B-F) The relative protein level of p-Met (B), N-cadherin (C), E-cadherin (D), Vimentin (E) and NGF (F) normalized to the ACTIN after treatment with PHA-665752 in PANC-1 cells; (G) Western blot analysis of the expression of p-Met, N-cadherin, E-cadherin, Vimentin and NGF at different PHA-665752 concentrations in BxPC-3 cells；(H-L) The relative protein level of p-Met (H), N-cadherin (I), E-cadherin (J), Vimentin (K) and NGF (L) normalized to the ACTIN after treatment with PHA-665752 in BxPC-3 cells.

**Figure S5**


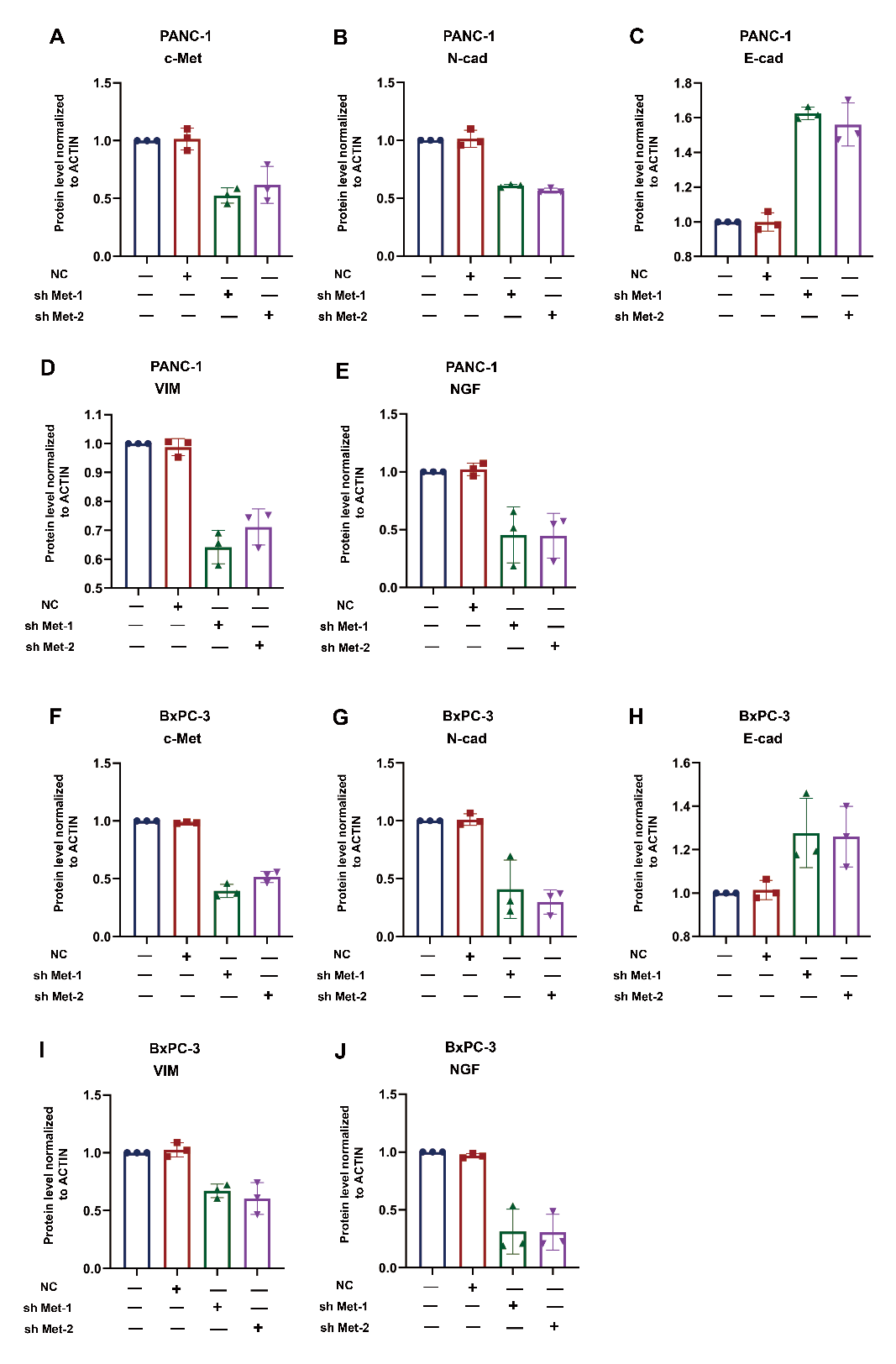


**Figure S5** (A-E) The relative protein level of c-Met (A), N-cadherin (B), E-cadherin (C), Vimentin (D) and NGF (E) normalized to the ACTIN in the wild-type, NC, sh Met-1, and sh Met-2 groups in PANC-1 cells; (F-J) The relative protein level of c-Met (F), N-cadherin (G), E-cadherin (H), Vimentin (I) and NGF (J) normalized to the ACTIN in the wild-type, NC, sh Met-1, and sh Met-2 groups in BxPC-3 cells.

**Figure S6**


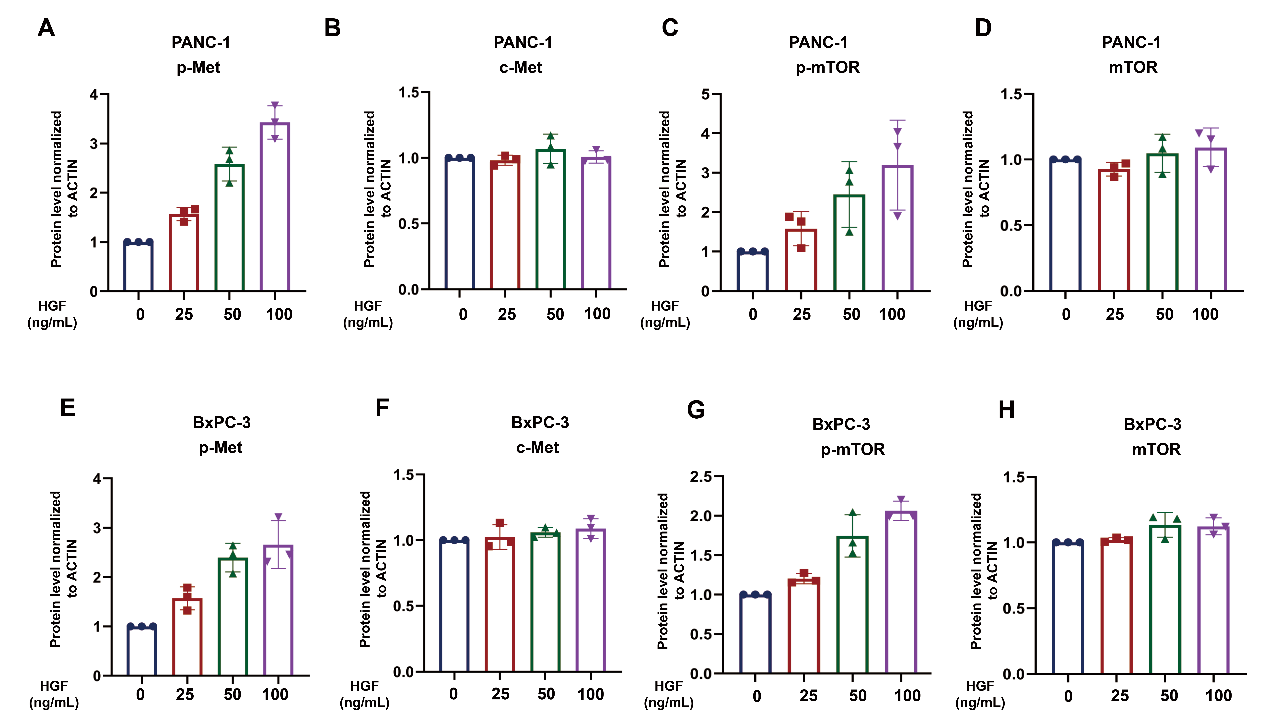


**Figure S6** (A-D) The relative protein level of p-Met (A), c-Met (B), p-mTOR (C) and mTOR (D) normalized to the ACTIN after treatment with HGF in PANC-1 cells; (E-H) The relative protein level of p-Met (E), c-Met (F), p-mTOR (G) and mTOR (H) normalized to the ACTIN after treatment with HGF in BxPC-3 cells.

**Figure S7**


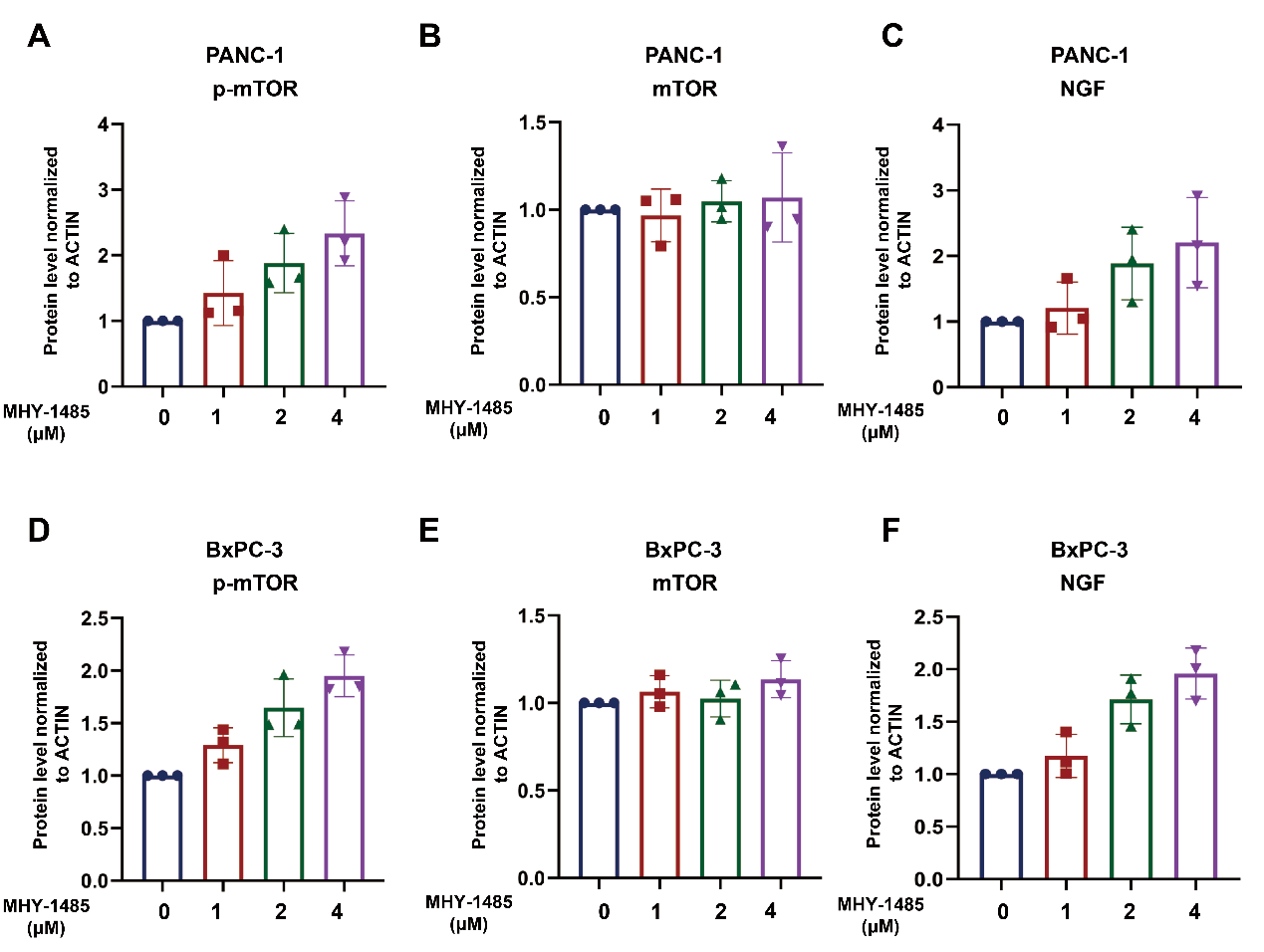


**Figure S7** (A-C) The relative protein level of p-mTOR (A), mTOR (B) and NGF (C) normalized to the ACTIN after treatment with MHY-1485 in PANC-1 cells; (D-F) The relative protein level of p-mTOR(D), mTOR (E) and NGF (F) normalized to the ACTIN after treatment with MHY-1485 in BxPC-3 cells.

**Figure S8**


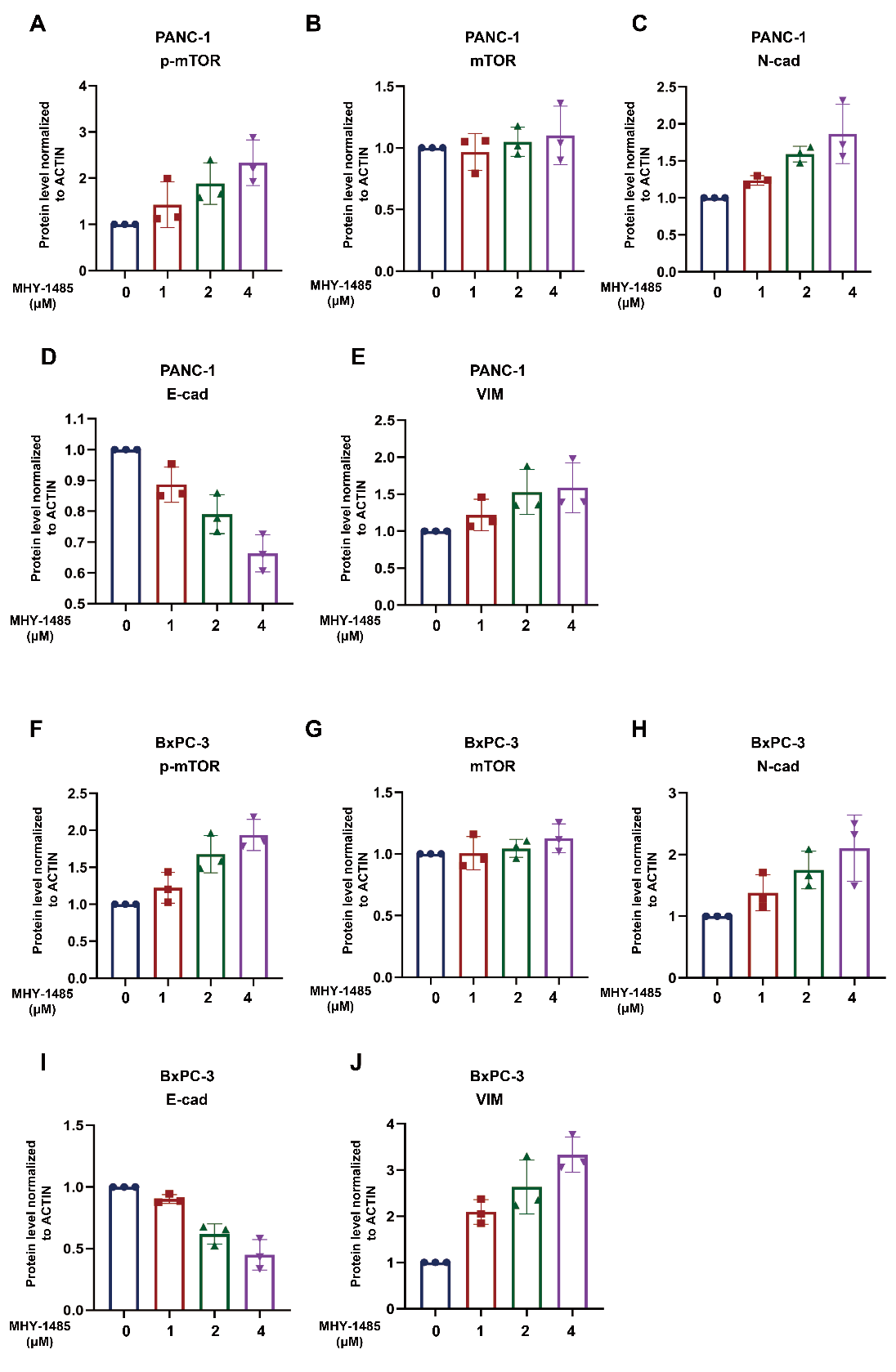


**Figure S8** (A-E) The relative protein level of p-mTOR (A), mTOR (B), N-cadherin (C), E-cadherin (D) and Vimentin (E) normalized to the ACTIN after treatment with MHY-1485 in PANC-1 cells; (F-J) The relative protein level of p-mTOR (F), mTOR (G), N-cadherin (H), E-cadherin (I) and Vimentin (J) normalized to the ACTIN after treatment with HGF in BxPC-3 cells.

**Figure S9**


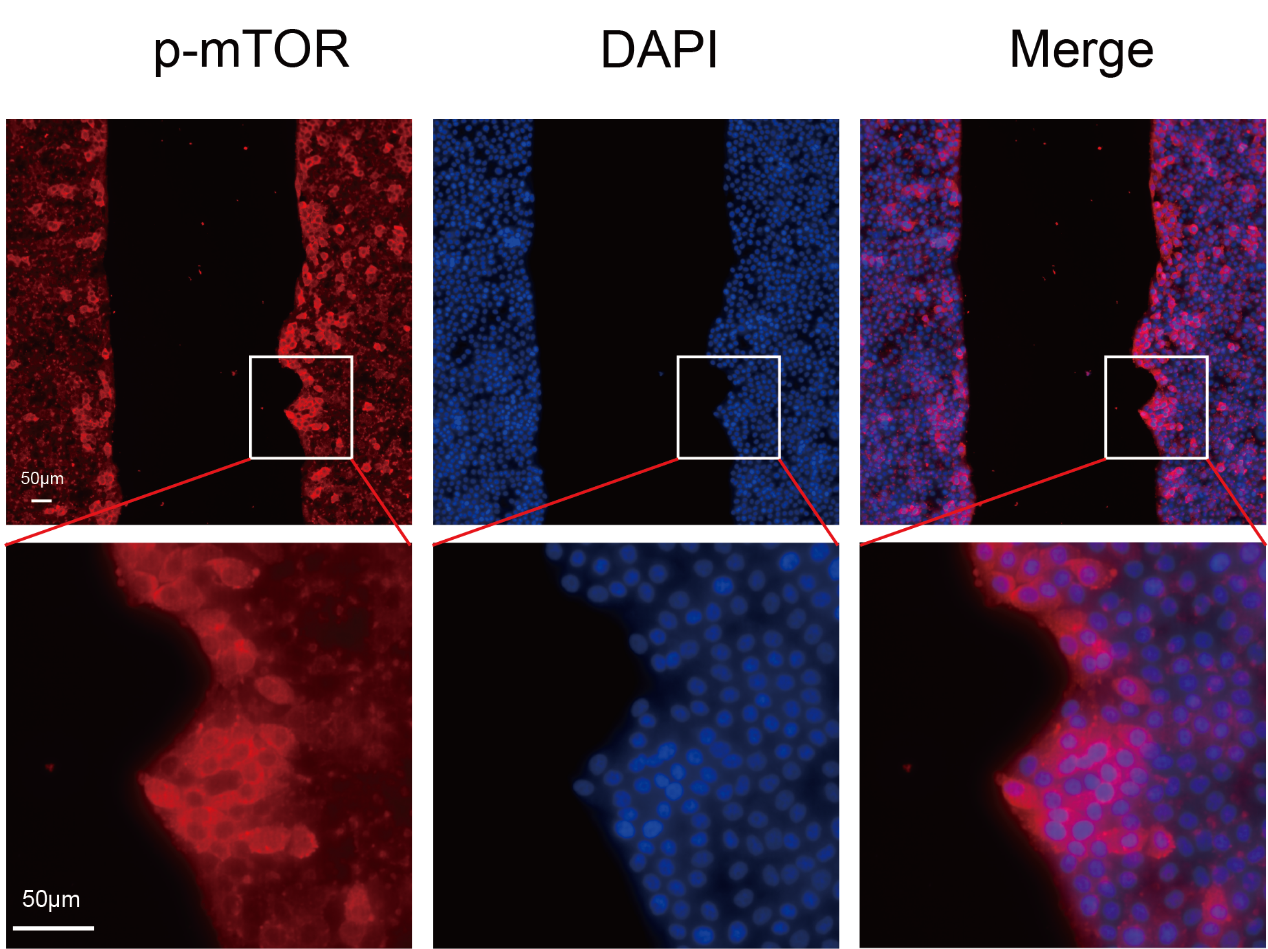


**Figure S9** Immunofluorescence staining for wound healing assay after treatment with MHY-1485 shows that the expression of p-mTOR of BxPC-3 cells that migrated to the gap front is higher.

**Figure S10**

**
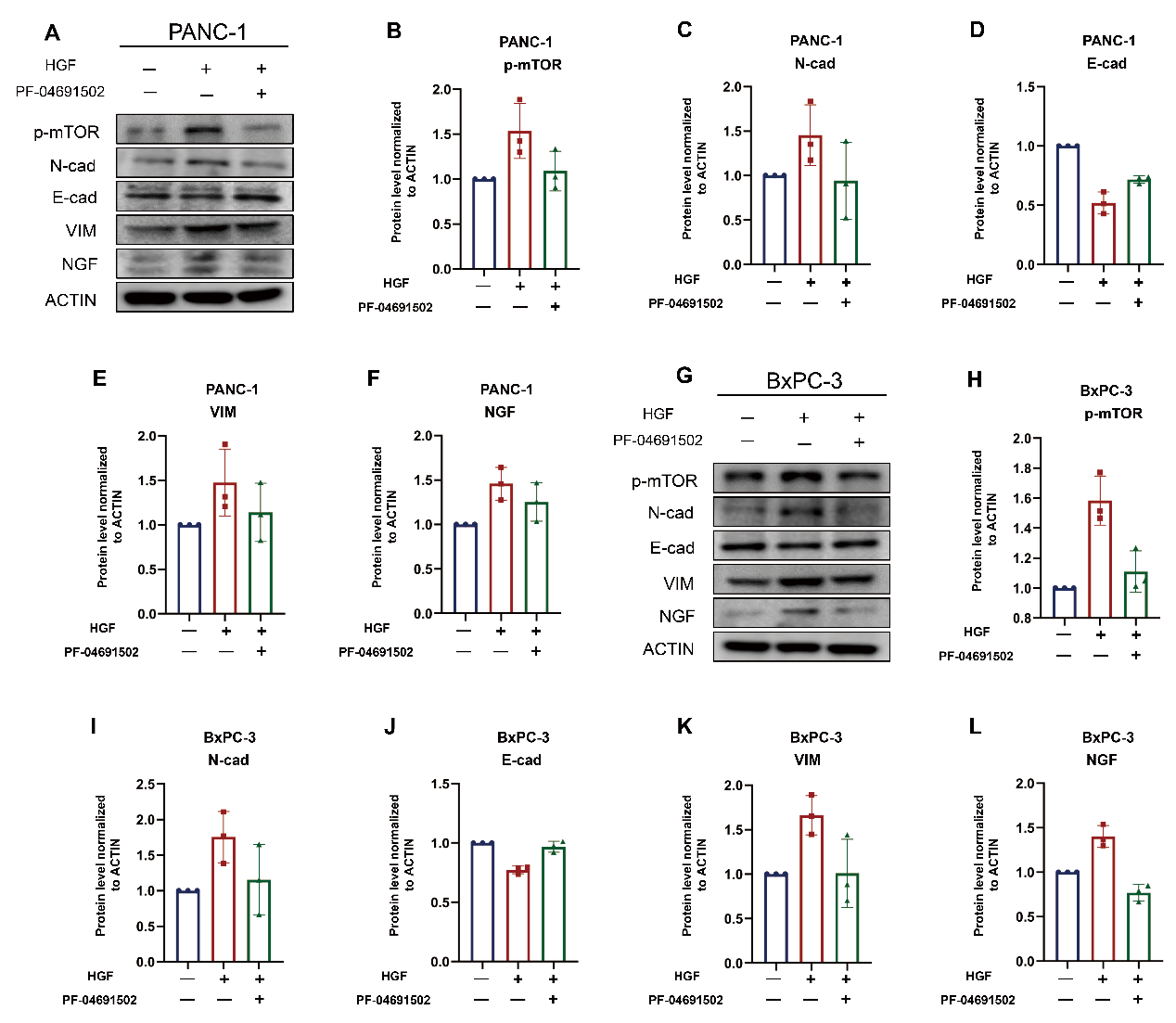
**

**Figure S10** (A)Western blot analysis of the expression of p-mTOR, N-cadherin, E-cadherin, Vimentin, and NGF in the control, HGF, and HGF+PF04691502 groups in PANC-1cells; (B-F) The relative protein level of p-mTOR (B), N-cadherin (C), E-cadherin (D), Vimentin (E) and NGF (F) normalized to the ACTIN in the control, HGF, and HGF+PF04691502 groups in PANC-1cells; (G)Western blot analysis of the expression of p-mTOR, N-cadherin, E-cadherin, Vimentin, and NGF in the control, HGF, and HGF+PF04691502 groups in BxPC-3 cells; (H-L) The relative protein level of p-mTOR (H), N-cadherin (I), E-cadherin (J), Vimentin (K) and NGF (L) normalized to the ACTIN in the control, HGF, and HGF+PF04691502 groups in BxPC-3 cells.

**Figure S11**


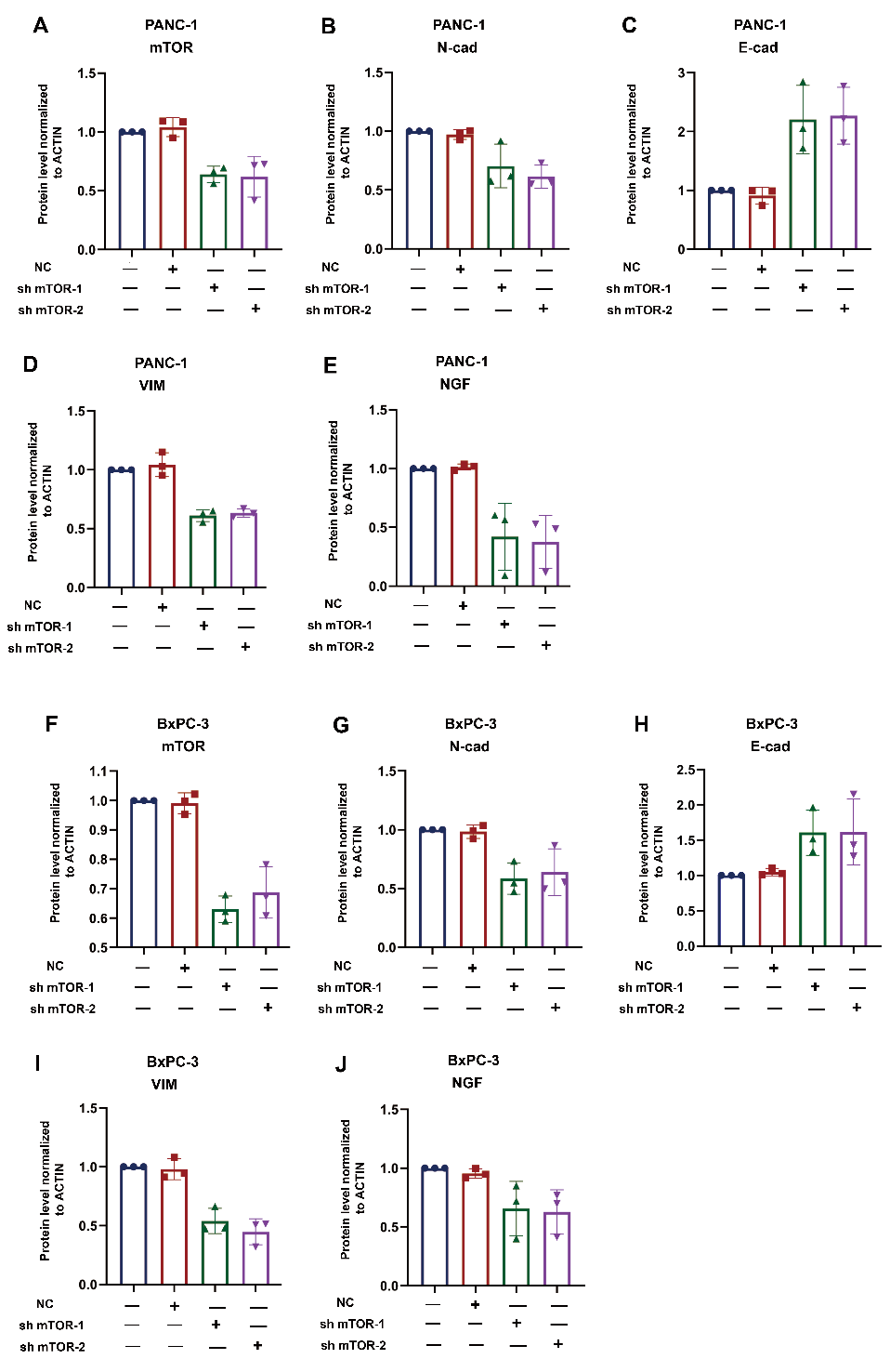


**Figure S11** (A-E) The relative protein level of mTOR (A), N-cadherin (B), E-cadherin (C), Vimentin (D) and NGF (E) normalized to the ACTIN in the wild-type, NC, sh mTOR-1, and sh mTOR-2 groups in PANC-1 cells; (F-J) The relative protein level of mTOR (F), N-cadherin (G), E-cadherin (H), Vimentin (I) and NGF (J) normalized to the ACTIN in the wild-type, NC, sh Met-1, and sh Met-2 groups in BxPC-3 cells.

**Figure S12**

**
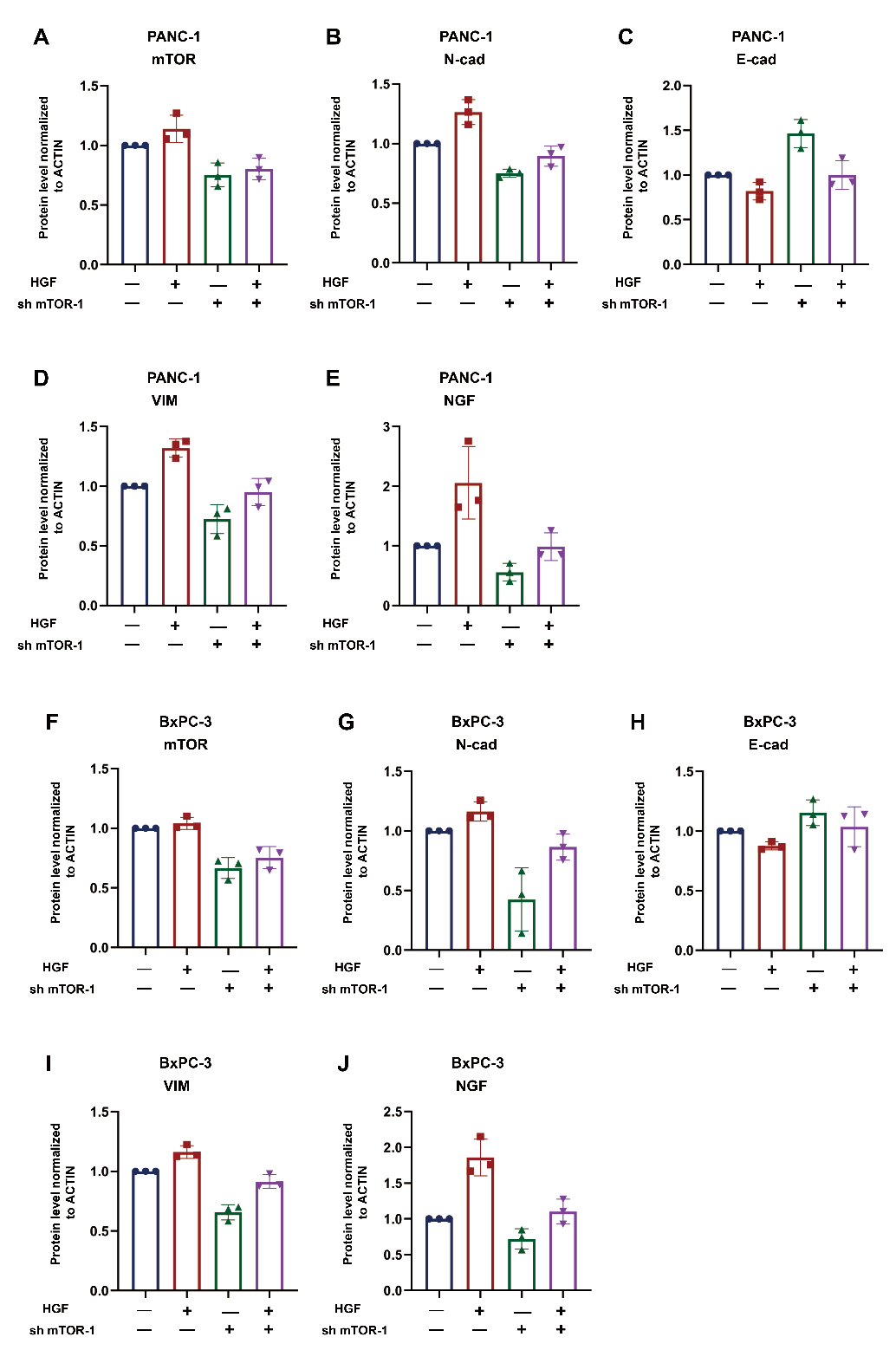
**

**Figure S12** (A-E) The relative protein level of mTOR (A), N-cadherin (B), E-cadherin (C), Vimentin (D) and NGF (E) normalized to the ACTIN in the NC, HGF, sh mTOR-1, and sh mTOR-1+HGF groups in PANC-1 cells; (F-J) The relative protein level of mTOR (F), N-cadherin (G), E-cadherin (H), Vimentin (I) and NGF (J) normalized to the ACTIN in the NC, HGF, sh mTOR-1, and sh mTOR-1+HGF groups in BxPC-3 cells.

**Figure S13**

**
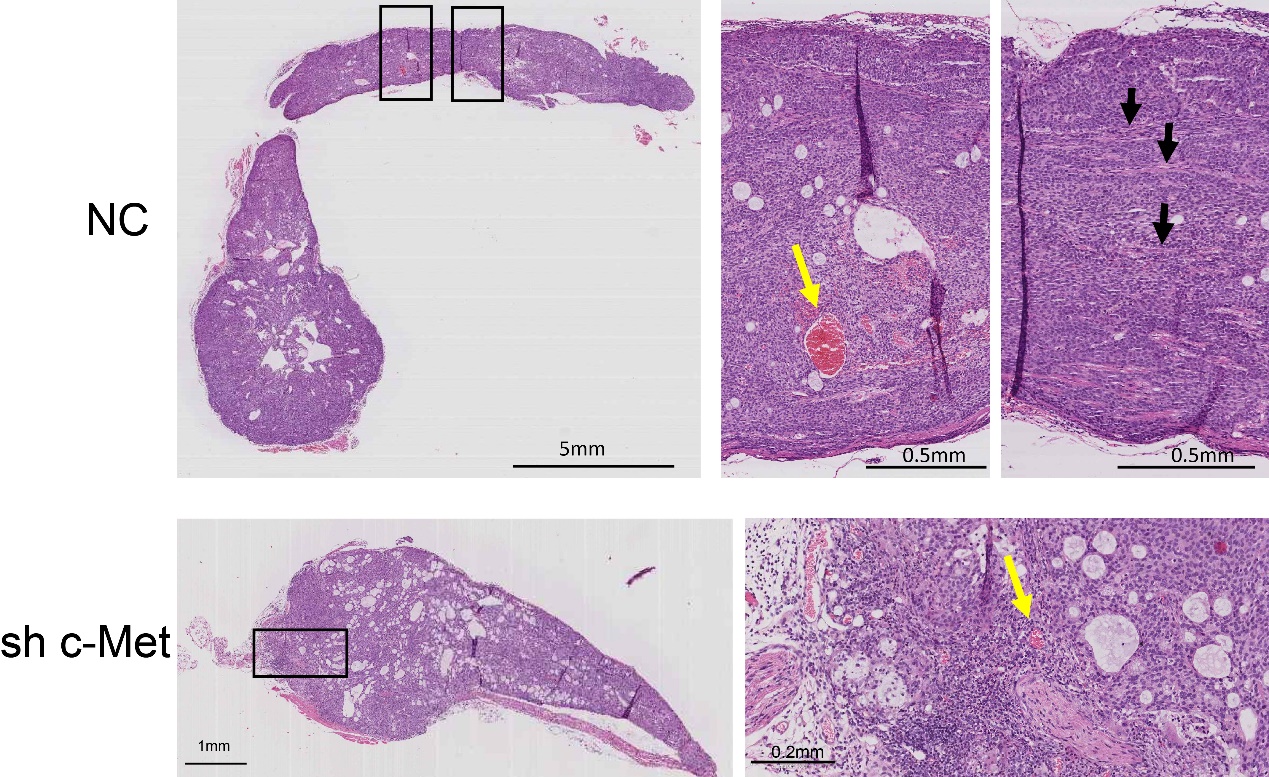
**

**Figure S13** HE staining showed cancer cells with high expression of c-Met have a richer blood supply in the sciatic nerve (yellow arrow). The black arrow indicates the nerve tissue.

**Figure S14**


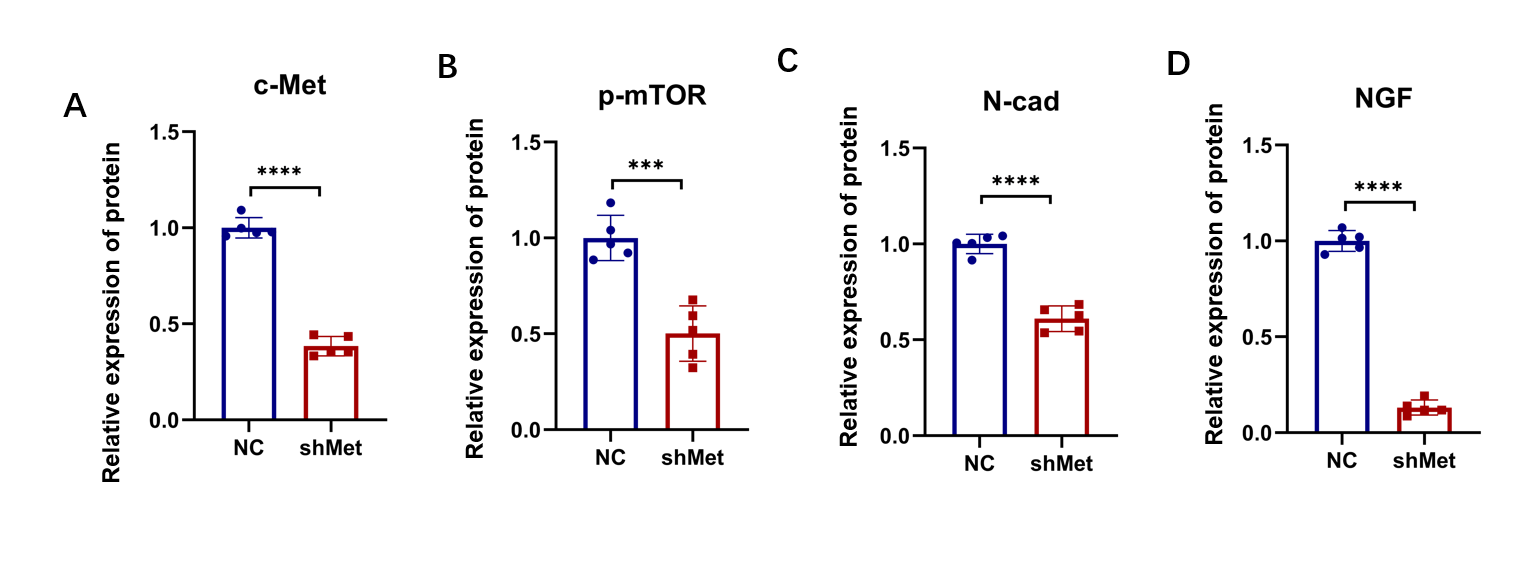


**Figure S14** (A-D) The relative expression of c-Met (A), p-mTOR (B), N-cadherin (C), and NGF (D) was detected by IHC in the NC and sh Met group. ****p*<0.001, *****p*<0.0001.

**Figure S15**

**
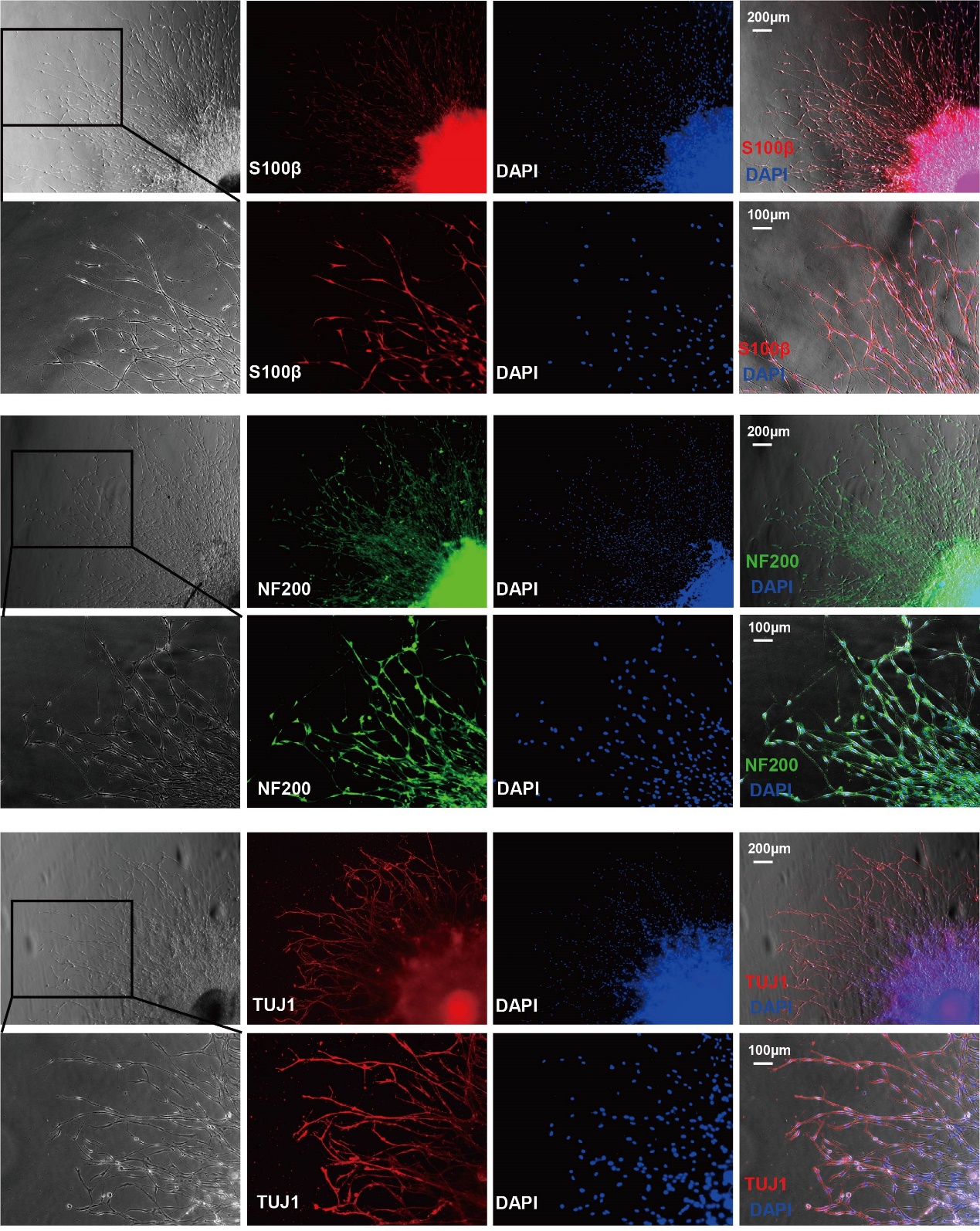
**

**Figure S15** The markers of S100β, NF-200, TUJ1 were used to identify new nerve axons.

**Figure S16**

**
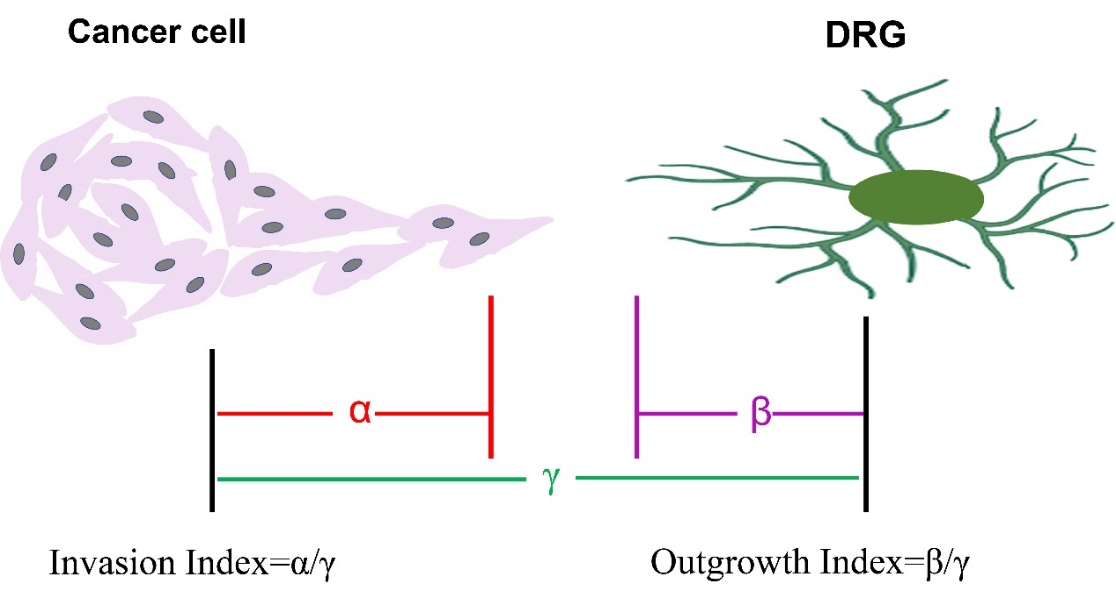
**

**Figure S16** The invasion index was used to assess cancer cell migration and invasion ((invasion index= α/γ), and the growth index was used to evaluate the ability of dorsal root ganglion axons to grow toward cancer cells （outgrowth index=β/γ）.
